# The Distribution of Fruit and Seed Toxicity during Development for Eleven Neotropical Trees and Vines in Central Panama

**DOI:** 10.1371/journal.pone.0066764

**Published:** 2013-07-02

**Authors:** Noelle G. Beckman

**Affiliations:** Ecology, Evolution, and Behavior, University of Minnesota – Twin Cities, Saint Paul, Minnesota, United States of America; The University of Texas at San Antonio, United States of America

## Abstract

Secondary compounds in fruit mediate interactions with natural enemies and seed dispersers, influencing plant survival and species distributions. The functions of secondary metabolites in plant defenses have been well-studied in green tissues, but not in reproductive structures of plants. In this study, the distribution of toxicity within plants was quantified and its influence on seed survival was determined in Central Panama. To investigate patterns of allocation to chemical defenses and shifts in allocation with fruit development, I quantified variation in toxicity between immature and mature fruit and between the seed and pericarp for eleven species. Toxicity of seed and pericarp was compared to leaf toxicity for five species. Toxicity was measured as reduced hyphal growth of two fungal pathogens, *Phoma sp.* and *Fusarium sp.*, and reduced survivorship of brine shrimp, *Artemia franciscana,* across a range of concentrations of crude extract. I used these measures of potential toxicity against generalist natural enemies to examine the effect of fruit toxicity on reductions of fruit development and seed survival by vertebrates, invertebrates, and pathogens measured for seven species in a natural enemy removal experiment. The seed or pericarp of all vertebrate- and wind-dispersed species reduced *Artemia* survivorship and hyphal growth of *Fusarium* during the immature and mature stages. Only mature fruit of two vertebrate-dispersed species reduced hyphal growth of *Phoma*. Predispersal seed survival increased with toxicity of immature fruit to *Artemia* during germination and decreased with toxicity to fungi during fruit development. This study suggests that fruit toxicity against generalist natural enemies may be common in Central Panama. These results support the hypothesis that secondary metabolites in fruit have adaptive value and are important in the evolution of fruit-frugivore interactions.

## Introduction

Selection pressure of mutualists and antagonists has resulted in a myriad of plant adaptations, from rewards for seed dispersers [1], [2] to defenses against herbivores, seed predators, and pathogens [3], [4]. The production of secondary metabolites is among one of the most important strategies that plants employ to mediate interactions with other organisms. Secondary metabolites are defined as compounds with no known physiological or primary metabolic functions. A number of these secondary metabolites have been found to function in plant defense [5]. In reproductive structures, specifically ripe fruit, secondary metabolites may also have additional functions in mediating interactions with seed dispersers [4]. The mediation of plant-animal and plant–microbe interactions by secondary compounds are known to influence plant survival and species distributions and are hypothesized to contribute to the generation and maintenance of plant diversity ([5] and references therein, [6], [7]).

The functions of secondary metabolites in plant defenses have been well-studied in green tissues [5], but not in reproductive structures [8]. Existing hypotheses outline the costs and benefits of allocation to chemical anti-herbivore defense in green tissue (reviewed in [9]). These hypotheses may be extended to the role of secondary metabolites in reproductive structures, as consumers of these structures have similar or potentially greater negative impacts on plant fitness [4], [10]. In plants dispersed by animals, toxicity of ripe pulp is viewed as a paradox, because pulp functions to attract mutualist seed dispersers [4]. However, ripe pulp attracts not only seed dispersers, but also consumers that have detrimental effects on fitness; various hypotheses have proposed these opposing selective pressures may explain the evolution of toxicity in ripe fruit [4], [11]. Alternatively, the presence of secondary metabolites in ripe pulp may not be adaptive but result from the plant’s general defense in leaves and immature fruit and the inability of the plant to reabsorb secondary compounds when the fruit ripens [10]. However, there is evidence suggesting that fruit chemistry is not constrained by leaf chemistry [12]. Furthermore, recent studies of *Capsicum* fruits suggest an adaptive function of secondary metabolites in the defense and dispersal of seeds [7], [13] and a trade-off between physical and chemical defense of chili fruits [13].

There is a dearth of studies on patterns of chemical defenses within reproductive structures and across developmental stages of fruit within natural communities [8]. The few studies of plants in natural communities have found inconsistent patterns of allocation to secondary metabolites throughout fruit development [14], [15], within fruits [16]–[18], and between vegetative and reproductive structures [17], [18]. Because understanding the evolution of frugivore-plant interactions has motivated the study of fruit chemistry, research has focused primarily on fleshy fruit, with fewer studies investigating allocation of chemical defenses in abiotically-dispersed species (*e.g.* [19]). Studies thus far have compared the concentrations of classes of compounds that are known to contribute to defense among different plant structures, but few studies test their direct role in defense against seed consumers [20], [21]. Because many compounds have synergistic effects and consumers from different taxa vary in their responses to secondary metabolites, it is important to determine the combined effect of these compounds on natural enemies [22]. Also, many plants have a diversity of secondary metabolites that may either be distributed throughout the plant or occur only in specific structures or locations, independently of each other [23], therefore quantifying the distribution of only a single class of compounds within plant structures isolated from the rest of the plant may not give an adequate representation of the total allocation of defenses distributed throughout the plant.

Variation in fruit defenses, including morphology and secondary compounds, may help explain interspecific variation in seed survival [24]. Many ecological studies in natural communities have examined the influence of seed size and other morphological fruit and seed traits on seed survival (*e.g.* [24]–[26]), while few have looked at the influence of fruit toxicity or how it may interact with morphology. Seed size is negatively correlated with pathogen attack [24], positively correlated with the size of mammals consuming seeds [27], and predicted to be positively correlated with insect seed predation; however, empirical support for this prediction remains unclear [26], [28]. Physical defenses, including the endocarp and testa surrounding the seed, have also been predicted to reduce natural enemy attack [29], and have been found to increase with seed size across species [28]. Physical defense was negatively correlated with chemical defense in one species of wild chili fruit polymorphic for the production of capsaicin, the chemical responsible for its pungency [13].

In this study, I examined the distribution of toxicity in eleven tropical forest tree and vine species using bioassays. Focal species included both vertebrate- and wind-dispersed taxa (Table 1). Instead of comparing specific known classes of defensive compounds (*i.e.* tannins, phenols, *etc*.), I determined whether plant extracts reduced the growth or survival of bioassay organisms compared to controls. Bioassays are a cost-effective method commonly used to screen for toxicity and integrate effects over unknown bioactive compounds [30], [31]. Using these bioassay data, the influence of fruit toxicity on seed survival in the presence of natural enemies was determined using data from a previous study [26]. With this study, I addressed the following questions and predictions:

**Table 1:** Study species.

Family	Genus Species	Site**	Life form†	Dispersal Mode†	Seed size (mg)‡	# Individuals
Anacardiaceae	*Anacardium excelsum* *	PNM	Tree	Mammal, Bat	1459.1	3
Rubiaceae	*Antirhea trichantha* *	PNM	Tree	Bird	1.5	3
Convolvulaceae	*Bonamia trichanta* *	PNM	Vine	Wind	16.1	1
Moraceae	*Castilla elastica* *	PNM	Midstory Tree	Mammal, Bird	203.3	3
Cecropiaceae	*Cecropia longipes*	PNM	Midstory Tree	Mammal, Bat, Bird	0.5	1
Cecropiaceae	*Cecropia peltata* *	PNM	Midstory Tree	Mammal, Bat, Bird	0.5	3
Bignoniaceae	*Jacaranda copaia*	PNSL	Tree	Wind	1.7	1
Tiliaceae	*Luehea seemannii* *	PNM	Tree	Wind	0.8	3
Lauraceae	*Nectandra umbrosa*	PNSL	Understory	Mammal, bird	276.5	1
Malpighiaceae	*Stigmaphyllon hypargyreum* *	PNM	Vine	Wind	9.8	1
Anacardiaceae	*Tapirira guianensis*	PNSL	Tree	Mammal, bird	183.3	3

† S.J. Wright personal communication,

‡ Seed reserve mass,

* Included in natural enemy removal experiment.

** Young leaves of five species were collected from the following locations in Central Panama: *Anacardium excelsum* and *Cecropia longipes* from Barro Colorado Island, *Tapirira guianensis* from Chagres National Park, *Bonamia trichantha* from Camino de Cruces National Park, and *Stigmaphyllon hypargyreum* form Coiba National Park (Coley & Kursar, pers. comm.).

1. Which plant parts are most toxic?
  - Is the seed or pericarp of mature fruit more toxic? For vertebrate-dispersed species, but not wind-dispersed species, the mature pericarp, or pulp in this case, attracts dispersers and should be lower in toxicity than the mature seed.
  - During which developmental stage are fruits most toxic? For vertebrate-dispersed species, but not wind-dispersed species, mature fruit are expected to be less toxic than immature fruit, and toxicity is expected to decrease more for the pericarp than the seed from the immature to the mature stage.
  - Are fruit or young leaves more toxic? If chemical defenses in fruit are a consequence of general defense of the plant, then fruit would be expected to have similar activity to leaves, which would decline with maturity in fruit of animal-dispersed species. However, fruit may have higher toxicity than green tissue as it contains nutrient packed seeds that are the plant’s direct link to future generations.
2. Is toxicity related to fruit morphology, specifically seed size and physical protection of the seed, independent of dispersal mode? Chemical defense may trade-off with physical defense as a method of protecting reproductive structures. Alternatively, plant species may vary in their defensive investments in general depending on their life history strategy, with high-defense species investing more in both physical and chemical defense.
3. Does fruit toxicity help explain variation in fruit development and seed survival in the presence of seed predators and pathogens? Increased toxicity to bioassay organisms would be expected to reduce damage by generalist natural enemies and therefore increase fruit development and seed survival.

## Methods

To determine patterns of chemical defense within species, I quantified the activity of extracts from different fruit parts and young leaves against invertebrates and fungal pathogens. Plant extracts were prepared from seed (*i.e.* embryo, endosperm, and testa) and pericarp of immature and mature fruit of eleven canopy species. The pericarp included the pulp for fleshy fruits of seven vertebrate-dispersed species and the capsule for three of the four wind-dispersed species included in this study. Crude extracts from young leaves of five of the study species were provided by T.A. Kursar and P.D. Coley. If extracts inhibited growth or survival of bioassay organisms, I refer to them as being toxic or having inhibitory activity; toxicity and inhibition therefore increase with decreasing growth or survivorship of bioassay organisms.

### Ethics Statement

The Smithsonian Tropical Research Institute and La Autoridad Nacional del Ambiente (Terrestrial Research Permit #SE/P-54-07) approved this study and gave permission to conduct research in Parque Natural Metropolitano and Parque San Lorenzo.

### Study Site and Species

Fruits were collected from Parque Natural Metropolitano (PNM), a dry, semi-deciduous, secondary forest located near the Pacific coast, and Parque Nacional San Lorenzo (PNSL), a wet, evergreen, old-growth forest located near the Atlantic coast in Central Panama using canopy cranes [32]. PNM consists of 265 ha of 80-year-old forest with trees reaching up to 40 m [32]. PNSL consists of 9600 ha of potentially 300-year-old forest (in the immediate surroundings of the crane) and secondary forest of varying ages with trees reaching up to 45 m [32]. Average annual rainfall at PNM and PNSL are 1740 and 3300 mm, respectively. The dry season in Central Panama begins in mid-December and lasts until the end of April. The canopy cranes in PNM and PNSL reach 42 and 52 m in height and cover an area of approximately 1 ha each, providing access to 80 and 180 species, respectively.

Eleven species were chosen to include a range of life forms, dispersal modes, and families based on the availability of fruit from reproductive individuals accessible from each crane (Table 1). Species from nine families included vines, understory, midstory, and canopy trees that were either wind- or vertebrate-dispersed. These species represent the community of fruiting individuals available to frugivores throughout the study period in proximity to each canopy crane. Each vine species in each study area was assumed to comprise one individual. Mean seed size ranged from 0.5 mg in *Cecropia longipes* and *C. peltata* to 1459 mg in *Anacardium excelsum*.

### Plant Collection and Processing

The timing of fruit collection depended on the fruiting season of each species and took place between March 2008 and September 2009. Fruits were monitored weekly to determine their development stage. Mature fruits were collected when ripe; immature fruits were collected approximately halfway through development. Depending on fruit and seed size, five to 500 fruits were collected from one to three trees and combined from all trees for extraction. Young leaves were collected from one individual of each species located in Central Panama between 1998 and 2004 (Coley & Kursar, pers. comm., Table 1). Because secondary compound concentrations and compositions can shift with leaf age [33], I focused on young leaves, whose defensive traits are predicted to be under stronger selection than those of mature leaves [34].

After collection, fruits were placed in sealed plastic bags on ice and processed in the lab the same day. If same-day processing was not possible, fruits were stored in ethanol or put in the freezer and processed within three days after sampling. Fruits were separated into seed and pericarp prior to processing when possible. For small-seeded, wind-dispersed species (*Bonamia trichantha, Jacaranda copaia,* and *Luehea seemannii*), fruits were separated into diaspore and capsule. For small-seeded, vertebrate-dispersed species (*Cecropia longipes* and *Cecropia peltata*), fruits were separated into diaspore and pulp. For ease of discussion, I will subsequently refer to all extracts from the seed or diaspore as seed extracts, and those from the pericarp, capsule, or pulp as pericarp extracts. For immature fruits of *Antirhea trichantha, Cecropia longipes, Cecropia peltata,* and *Jacaranda copaia*, seeds were too undeveloped to separate from the pericarp, and therefore the entire fruit was used for the extractions. Fruits were weighed and either used for extractions or put into a drying oven to obtain dry weights of the seed reserve (*i.e.* embryo and endosperm) and physical defense per diaspore (*i.e.* dry mass of endocarp and testa/dry mass of diaspore). To obtain dry masses, fruits were placed in a drying oven at 60°C for at least 72 h.

To prepare the crude extract, fruit material was macerated in methanol using a Waring blender and then a Polytron homogenizer (Brinkmann Instruments). A mortar and pestle were used to macerate seeds. The marc, or components of the fruit that remained following extraction, was then washed with approximately 1/2 to an equal amount of ethyl acetate (depending on the species), and filtered successively under vacuum through Whatman #4 and #1 filter paper. The marc was washed one to two more times in methanol and ethyl acetate until all soluble compounds were extracted (*i.e.* the solvent remained clear after washing the marc); this resulted in approximately 0.03–0.3 total solvent (ml) per fruit dry mass (mg) depending on the extract. Extracts of young leaves were prepared similarly with methanol and ethyl acetate (Coley & Kursar, pers. comm.). The combined fractions of methanol and ethyl acetate from fruit and leaf extracts were concentrated using a rotary evaporator at 40°C, freeze-dried, and stored at −80°C. Before conducting bioassays, extracts were redissolved in the combined solvents of methanol and ethyl acetate (the exact proportions depended on the species) for the preparation of a range of concentrations. Percent extract per fruit part was estimated using extract dry mass per amount of fruit part collected and a conversion factor for the amount of fruit part collected to the average fruit part dry mass.

### Secondary Metabolite Bioassays

I conducted three different bioassays, using an invertebrate (*Artemia*) and two fungal pathogens (*Fusarium sp*. and *Phoma sp.*). The brine shrimp test is a general bioassay that was developed as a pre-screening for cytotoxicity to aid in drug discovery and evaluate toxicity of natural pesticides against insects [35]. Methods for the brine shrimp bioassay were modified from Solis et al. [36]. *Artemia franciscana* cysts (Ocean Star International, Red Jungle Brand from Aquarium World, Panama City, Panama) [37] were hatched in 3% sterile distilled seawater (3 g Instant Ocean® Sea Salt in 100 ml sterile deionized water) in a separatory funnel under constant light and aeration. After two days, larvae were removed from the funnel for use in bioassays. Crude extracts of fruits were replicated five times at three concentrations (0.01, 0.1, and 1 ug/ml). To prepare concentrations, the redissolved fraction was dispensed into 2 ml microcentrifuge tubes and evaporated under vacuum using a SpeedVac to remove the solvents. Then dimethylsulfoxide (DMSO) was added at 1% of the final volume to solubilize nonpolar compounds. Extracts were diluted with 3% seawater to obtain a concentration twice the final concentration. For each replicate, 100 ul of this dilution was dispensed into one well of a 48 Multiwell™ plate. Each plate also included five wells of a negative control consisting of 3% seawater and 1% DMSO (of the final volume). A positive control of acetic acid (CH_3_COOH) at 0.01, 0.05, 0.1, 0.5, 1 ul/ml was also included. To determine toxicity, 100 ul of seawater containing *ca.* 10–100 larvae was added to each well. Plates were covered and incubated in the dark at room temperature for 48 hours. After 24 and 48 hours, dead larvae were censused using a Motic stereoscope and classified as dead if unmoving for 10 seconds. Following the 48 hour census, 100 ul of methanol was added to all wells to kill all shrimp and the total number of shrimp in each well could then be counted accurately. For acetic acid, published results show a fifty percent reduction in *Artemia* survival at a concentration of 0.134 ul/ml after 24 hours at 3.5% salinity [38]. In my study, a fifty percent reduction in *Artemia* survival after 48 hours occurred at a concentration of 0.21 (±0.02 SE) ul/ml of acetic acid (see ‘Statistical analyses’ below).

**Figure 1:**
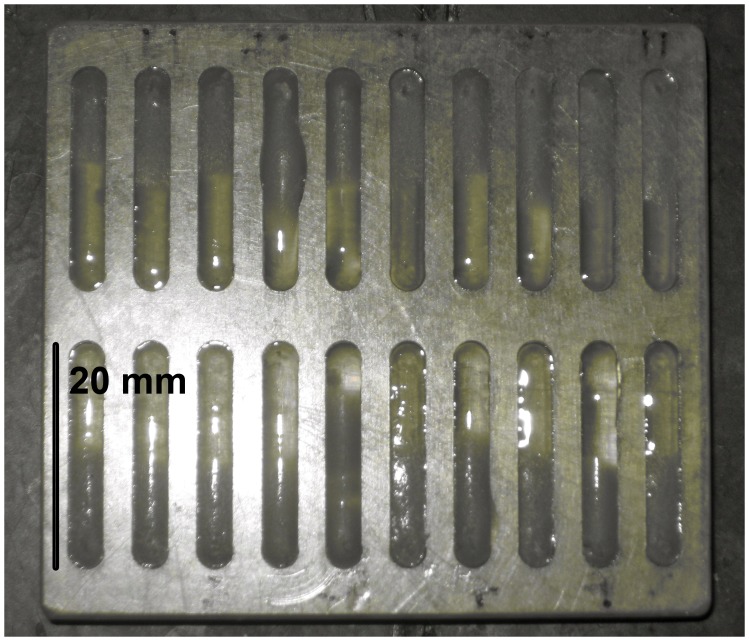
Twenty-lane plates used for fungal bioassays. (K.G. Murray, Hope College).

The two fungal pathogen bioassays tested how plant extracts inhibited two foliar fungal pathogens collected previously and archived in Panama (one isolate from *Phoma sp*. and *Fusarium sp*.) [39]. Species in these genera are important seed pathogens in a diverse collection of hosts [40]. Fungal bioassays of each crude plant extract were replicated five times at each concentration tested. Each extract was first tested at a concentration of 17% dry mass (100* mg extract/mg agar). If activity was found, extracts were then tested sequentially at 9.1, 4.8, 2.4 and 1.2% dry mass, discontinuing the series if no inhibition was found. Bioassays were conducted in standard size Petri dishes that contain plates with 20 lanes developed for bioassays of filamentous fungi requiring small volumes of media (Fig. 1, K.G. Murray, Hope College). In each plate, five replicates of three different extracts and a negative control were dispensed. Nadixic acid was used as a positive control at 0, 100, 200, 300, 400, and 500 ug/ml. Malt extract agar in 1% DMSO was mixed with plant extract mixtures after first evaporating solvent. Mixtures were kept in a water bath at 45°C for 30 minutes, vortexing several times to ensure proper mixing of the extract and agar prior to introduction to plates. For *Phoma,* plates were inoculated with a small plug of agar (3 mm diameter×5 mm thickness), and, for *Fusarium*, plates were point inoculated with hyphae (because *Fusarium* hyphae became aerial and infiltrated neighboring lanes within the petri dish when using plugs). Hyphal growth, a measure of effects on plant fitness [41], was measured using a Motic stereoscope after 48 hours for *Fusarium* and after 72 hours for *Phoma.* In the positive control, fifty-percent reduction in hyphal growth was found at 353 (±34 SE) mg/ml in the *Fusarium* bioassay and 387 (±12 SE) mg/ml in the *Phoma* bioassay (see ‘Statistical analyses’ below).

### Experimental Study of Seed Survival in Response to Natural Enemies

In a previous study, the effects of vertebrates, insects, and pathogens on fruit development and seed survival in the forest canopy were determined for seven tropical species (Table 1). In addition, the influence of morphological fruit traits on these interactions were quantified [26]. To determine the relative effects of vertebrates, insects, and pathogens on seed survival in the canopy, each of these organisms were experimentally removed from branches using exclosures, insecticides, and fungicides, respectively. Treatments included these main effects plus a control, in which no organisms were removed. Fruits were censused during development to determine the proportion that reached maturity, and germination trials were used to assess seed viability. One species, *Cecropia peltata*, was included only in the analysis of germination; it was not included in the analysis of fruit development because fruit removal was measured using a differed method than the other species.

For these seven species, the following morphological fruit traits were measured: pulp-to-fruit dry mass ratios, capsule-to-fruit dry mass ratios, physical defense per diaspore, log (mean fruit dry mass), log (mean fruit length), log (mean fruit width), log (mean seed reserve dry mass), and log (mean number of seeds); physical defense was calculated as seed reserve dry mass subtracted from diaspore dry mass [28]. Interspecific variation in fruit morphology was summarized with principal component analysis using standardized variables of traits (*i.e.* correlation matrix in the PCA) [14]. The first three principal components (PC1, PC2, and PC3) explained 91 percent of the variance and were included in subsequent analyses of fruit development and seed germination. The first principal component most strongly reflects fruit size (positively) vs. physical defense per diaspore (negatively), the second reflects seed size (positively) vs. seed number per fruit (negatively), and the third reflects the capsule-to-fruit ratio (positively) vs. the pulp-to-fruit ratio (negatively). For more detailed methods and results of this study, see Beckman and Muller-Landau [26].

### Statistical Analyses

***Which plant parts are toxic?*** I used two different approaches to determine toxicity of plant extracts to bioassay organisms. In the first approach, toxicity of the highest concentration of each plant extract relative to the negative control was quantified for each bioassay organism using a linear mixed modeling approach with extract identity as the fixed effect. For *Artemia* bioassays, the proportion of shrimp surviving in 1 mg/ml plant extract was modeled with generalized linear mixed models (GLMM) with binomial errors. Fungal hyphal growth was normally distributed in both *Fusarium* and *Phoma* bioassays, and the effect of 17% dry mass extracts on hyphal growth was modeled using a linear mixed model with normal errors. Plate number (*Artemia*) or Petri dish (fungal pathogen) was included as a random effect in the model. The coefficient estimates from these models represent the difference in the mean response of a bioassay organism in a particular extract relative to negative controls, and are used to describe the activity of each extract. Significant negative coefficient estimates were interpreted as more toxic than controls, zero or nonsignificant values indicated no toxic effect, and significant positive values indicated beneficial effects of plant extracts on bioassay organisms. The Laplace approximation of likelihoods was used to estimate coefficients for fixed- and random-effects using restricted maximum likelihood estimation [42]. Because of the uncertainty in calculating degrees of freedom needed for Wald *t* or *F*-tests in generalized linear mixed models with normal errors, calculating *P*-values is controversial [42]. As the sample size is large (*N* = 320 for *Artemia*, *N* = 315 for *Phoma*, *N* = 320 for *Fusarium* bioassays), the *t*-value was assumed to be approximately normally distributed, and the normal distribution was used to calculate *P*-values [43]. GLMM analyses were performed using the lme4 package in R [44], [45].

In the second approach, I determined the effective dosage that resulted in a 50% reduction in fungal hyphal growth or *Artemia* survivorship (ED_50_) by fitting curves of each response across concentrations of each extract using the ‘drc’ package in R [46]. Fungal responses to varying concentrations of each extract are expressed as hyphal growth relative to the mean hyphal growth of controls in each dish (GRC) [47]. A three-parameter Weibull model was fit to GRC across concentrations for each extract to estimate ED_50_ and its standard error. The three parameter model is given by f (C) = *a* exp (−exp [*b* (log (*C*)−*g*)]), where *C* is the concentration, *a* is the response at the highest concentration, *b* is the slope around *g*, and *g* is the logarithm of the inflection point. To determine the ED_50_ of the mature pericarp extract from *Anacardium excelsum* in the *Phoma* bioassay, a five parameter Brain-Cousens curve was fit. This curve allows for increased growth compared to controls at low concentrations and reduced growth at higher concentrations; it is given by f(C) = c+(d−c+ f*C)/[1+ exp (*b* [log (*C*) – log (g)])]. For extracts that significantly reduced *Artemia* survival, a two parameter logistic function given by f(*C*) = (1+ exp [*b* (log (*C*) −ED_50_ )])^−1^ was fit to binomial data weighted by the number of total shrimp to estimate ED_50_ and its standard error. The two parameter model assumes the highest and lowest survivorship are one and zero, respectively.

To determine the importance of dispersal mode, fruit developmental stage, and plant part in explaining variation in activity of plant extracts, I compared a suite of nested models using Akaike Information Criterion (AIC) values. For *Fusarium* and *Phoma*, I analyzed variation in hyphal growth on 17% dry mass plant extract divided by the mean hyphal growth on negative controls in each dish (GRC). Because GRC was normally distributed, I used a linear mixed model with a normal error distribution. Variation in *Artemia* survivorship in response to 1 mg/ml plant extracts was analyzed using a binomial error distribution; although *Artemia* survivorship in treatments was not adjusted relative to controls, survivorship in controls was high (96%). In addition to plate number of Petri dish, species nested within dispersal mode was included as a random effect to account for variation among species within dispersal modes. To compare linear mixed models, models differing in fixed effects were fit by maximum likelihood estimation and the most parsimonious model was refit with the restricted maximum likelihood method to estimate parameters. With this approach, I tested the following:

- *Is the seed or pericarp of mature fruit more toxic?* To test the prediction that the mature pericarp is lower in toxicity than the mature seed for vertebrate-dispersed seeds but not wind-dispersed seeds, I included dispersal mode (vertebrate/wind), fruit part (seed/pericarp), and their interaction as fixed effects using data for mature plant extracts only (Table 2).
- *During which developmental stage is fruit most toxic?* To test the prediction that mature fruit parts are less toxic than immature fruit parts and that the toxicity of the pericarp decreases more than the seed from the immature to the mature stage in vertebrate-dispersed species but not wind-dispersed species, I included the following predictors: dispersal mode (vertebrate/wind), fruit part (seed/pericarp), fruit developmental stage (immature/mature), and their interactions (Table 3). This analysis was done on a subset of seven species for which extracts of the seed and pericarp were available at both the immature and mature stages.
- *Are fruit or young leaves more toxic?* To test whether fruit or young leaves are more toxic, I included dispersal mode (vertebrate/wind), plant part (leaf/fruit), and their interaction as fixed effects (Table 4). This analysis was done on a subset of five species for which extracts from the fruit and leaves were available. In the model, leaves were compared to all available fruit extracts (*i.e.* immature seed, pericarp, or whole fruit, and mature seed or pericarp). To account for this pseudoreplication within species, I included plant part nested within species as a random effect.

**Table 2:** Is the seed or pericarp of mature fruit more toxic?

	AIC	
Terms in Model	*Artemia*	*Fusarium*
Dispersal Mode×Fruit Part	**1199.9**	**−83.9**
Dispersal Mode+Fruit Part	1536.3	**−83.8**
Fruit Part	1537.0	**−85.8**
Dispersal Mode	1537.4	−77.4
Null	1538.1	−79.4

*Notes:* Comparison of AIC values for generalized linear mixed effects models of *Artemia* survivorship in fruit extract and *Fusarium* hyphal growth on fruit extract relative to controls for mature fruit of eleven species. AIC values of models within two AIC of best-fit models are in bold.

**Table 3:** During which developmental stage is fruit most toxic?

	AIC	
Terms in Model	*Artemia*	*Fusarium*
Dispersal Mode×Fruit Part×Fruit Stage	**1452.7**	−48.5
Dispersal Mode×Fruit Part+Dispersal Mode×Fruit Stage+Fruit Part×Fruit Stage	1466.7	−49.8
Fruit Stage+Dispersal Mode×Fruit Part	1487.4	**−52.6**
Fruit Part+Dispersal Mode×Fruit Stage	1666.6	−42.3
Dispersal Mode+Fruit Part×Fruit Stage	1694.2	−37.7
Dispersal Mode+Fruit Part+Fruit Stage	1692.5	−38.3
Dispersal Mode×Fruit Part	1485.9	**−52.4**
Dispersal Mode+Fruit Part	1691.1	−37.3
Dispersal Mode×Stage	1689.6	−42.1
Dispersal Mode+Stage	1710.1	−35.5
Stage×Fruit Part	1692.2	−38.4
Stage+Fruit Part	1690.5	−38.9
Dispersal Mode	1708.8	−34.6
Fruit Part	1689.1	−37.9
Fruit Stage	1708.1	−36.0
Null	1706.8	−35.0

*Notes:* Comparison of AIC values for generalized linear mixed effects models of *Artemia* survivorship in fruit extract and *Fusarium* hyphal growth on fruit extract relative to controls for immature and mature fruit of seven species. AIC values of models within two AIC of best-fit models are in bold.

**Table 4:** Are fruit or young leaves more toxic?

	AIC	
Terms in Model	*Artemia*	*Fusarium*
Dispersal Mode×Plant Part	1226.2	−98.5
Dispersal Mode+Plant Part	1224.2	−98.5
Plant Part	**1222.3**	−**99.9**
Dispersal Mode	**1222.7**	**−100.4**
Null	**1220.8**	**−101.7**

*Notes:* Comparison of AIC values for generalized linear mixed effects models of *Artemia* survivorship in plant extract and *Fusarium* hyphal growth on plant extract relative to controls for fruit and leaves of five species. AIC values of models within two AIC of best-fit models are in bold.

Following this model comparison approach, I investigated variation in toxicity among fruit developmental stages and plant parts more fully. Using the activity values (coefficient estimates and standard errors) assigned from the model determining toxicity of extracts in each bioassay (from the first modeling approach under ‘*Which plant parts are toxic?*), I performed analyses with *a priori* contrasts within species (Supporting Information S1). Differences between immature seed and pericarp, mature seed and pericarp, immature and mature seed, immature and mature pericarp, and all fruit parts to young leaf extracts were tested (Table 5). Comparisons among all plant parts and developmental stages were performed within species because the best-fit model for variation in responses of each bioassay organism included the three-way interaction between species, fruit development stage, and fruit part for the subset of seven species that included all levels of fruit developmental stage and fruit parts (results not shown).

**Table 5:** *A priori* contrasts used to determine toxicity of extracts relative to controls and differences of extract toxicity within plant species following analyses of *Phoma* hyphal growth, *Fusarium* hyphal growth, and *Artemia* survivorship in response to plant extracts versus controls.

Variable 1	Variable 2
Control	All extracts
Immature seed or diaspore	Immature pericarp, capsule, or pulp
Immature seed, diaspore, or whole fruit	Mature seed or diaspore
Immature pericarp, capsule, pulp, or whole fruit	Mature pericarp, capsule, or pulp
Mature seed or diaspore	Mature pericarp, capsule, or pulp
Mature calyx (*Castilla elastica* only)	Mature seed or pericarp
Leaves	All fruit extracts

*Notes:* Plant extracts are only compared within plant species for each bioassay.

***Is toxicity related to fruit morphology (seed size and physical protection)*** **?** To determine whether there was a trade-off between chemical and physical defense, I tested for a correlation between activities of each fruit part at each stage and fruit morphology, specifically seed mass and physical defense (Pearson’s product moment correlation coefficient). Because four species did not have extracts from the immature seed or pericarp, the activity values of the immature whole fruit were used in place of these values.

***Does fruit toxicity help explain fruit development and seed germination?*** To assess the effect of toxicity on fruit development and germination, species-level mean toxicity values were included as covariates in analyses of fruit development and seed survival of seven species from a previous experiment [26]. To calculate mean toxicity, coefficient estimates of seed and pericarp extracts from immature fruits were averaged for *Artemia* and *Fusarium* bioassays. I did not use data from the *Phoma* bioassay in this analysis because immature fruit extracts did not inhibit *Phoma* growth. The first three principal components of fruit morphology were included in models as covariates to account for differences in fruit morphology previously shown to be important in explaining variation in fruit development and germination [26]. To account for spatial autocorrelation among seeds that were collected from the same branches and trees, branch nested within tree was included as a random effect. Akaike Information Criterion was used to select the most parsimonious model from a set of candidate models that differed in the inclusion of toxicity covariates and their interactions with natural enemy treatments. For further description of statistical methods and results for variation in fruit development and germination explained by fruit morphology, see Beckman and Muller-Landau [26]. All statistical analyses were done using R [45]. Data available from the Dryad Digital Repository: http://dx.doi.org/10.5061/dryad.b2c80 [48].

## Results

### Which Plant Parts are Toxic?

**Figure 2:**
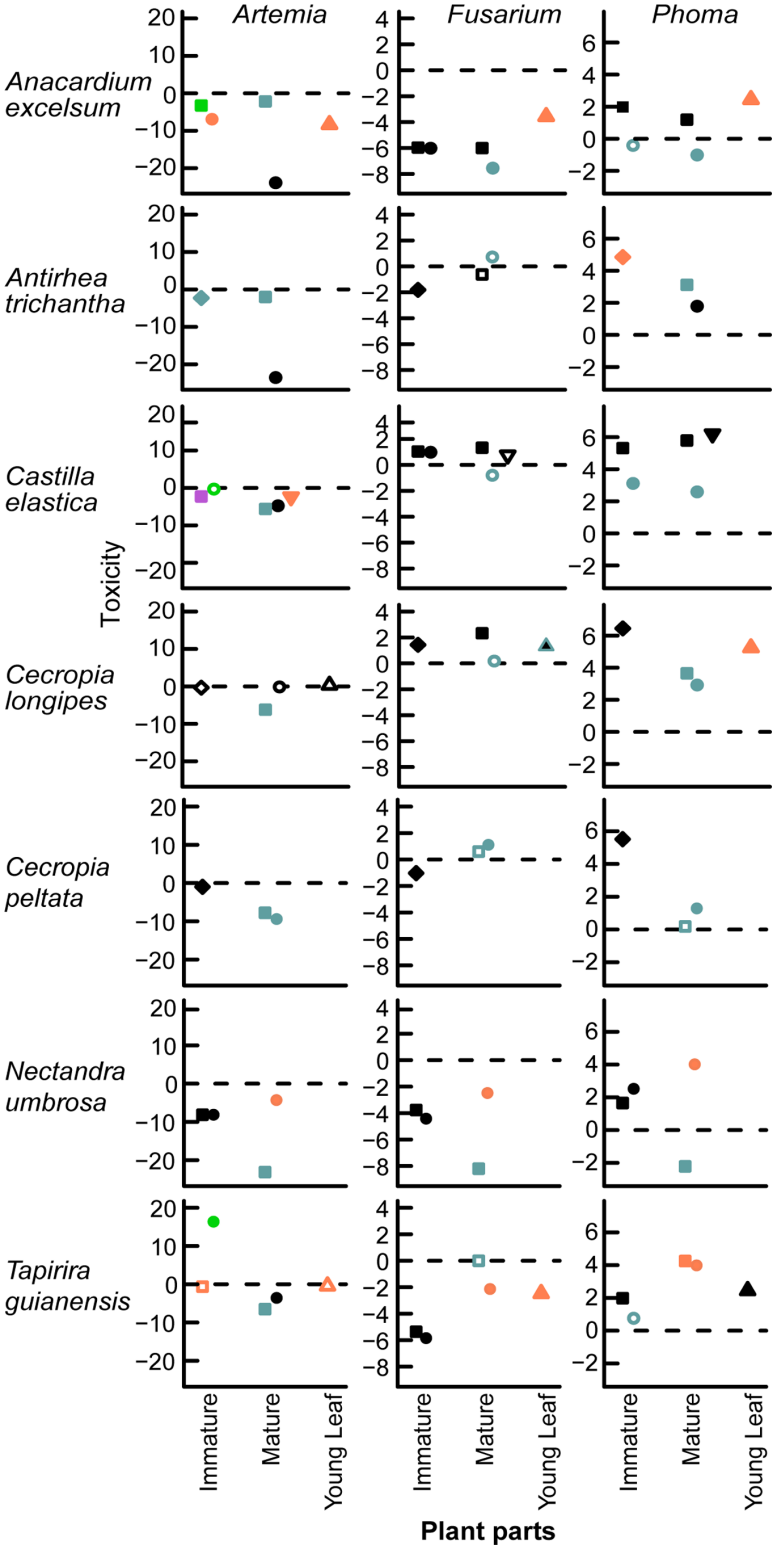
Toxicity of vertebrate-dispersed species. Shown are the coefficient estimates from generalized linear mixed models of the difference between the proportion of surviving brine shrimp *Artemia* and hyphal growth of *Fusarium* and *Phoma* on extracts relative to controls. Symbols designate the following plant parts: pericarp (•), seed (▪), whole fruit (□), leaf (▴), and calyx (**▾**). Species are ordered in increasing seed size. Coefficient estimates are differences in hyphal growth (mm) in plant extracts from negative controls for fungal bioassays, and the log of the odds ratio between plant extracts and negative controls for the brine shrimp bioassay. Values below zero indicate reduced survivorship or hyphal growth and values above zero indicate increased hyphal growth. Solid symbols indicate significant differences of responses of organisms in treatments compared to controls at the 0.05 significance level, whereas unfilled symbols indicate no significant differences from controls. Different colors indicate significant differences, while similar colors indicate no significant differences among means within species.

**Figure 3:**
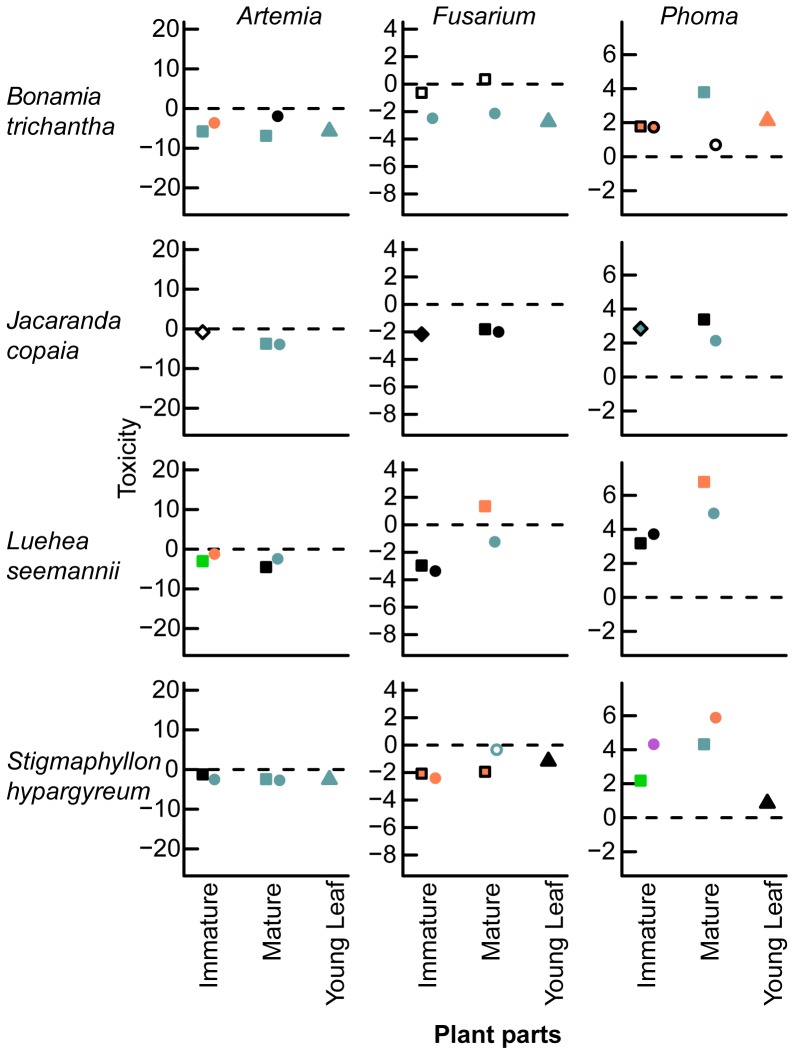
Toxicity of wind-dispersed fruit. See Fig. 3 caption for more details.

Every plant species had at least one extract that significantly inhibited at least one bioassay organism (Fig. 2, 3; Table 6). Nine plant species significantly inhibited two organisms and two plant species inhibited all three bioassay organisms. Ten of the eleven plant species had at least one immature seed or pericarp that was toxic to at least one bioassay organism, and all species had at least one mature fruit part that was toxic to at least one bioassay organism. The seed and pericarp of the majority of vertebrate- and wind-dispersed species reduced *Artemia* survivorship and hyphal growth of *Fusarium* during the immature and mature stages (Table 7). Only mature fruit of two vertebrate-dispersed species reduced hyphal growth of *Phoma*. For 26 of the toxic extracts, the ED_50_’s were higher than the concentrations that were tested in this study, however the concentrations generally found within fruit were within range of the ED_50_’s (Table 8). Because the majority of extracts were not toxic to *Phoma* (Fig. 2, 3), I did not analyze variation in extract activity using the model selection approach in the following sections.

**Table 6:** Which plant parts are toxic?

Species	Stage	Fruit Part	*Artemia* Coefficient Estimates at 1 mg/ml (SE), *N* = 320	*Fusarium* Coefficient Estimates at 17% mg/mg (SE), *N* = 320	*Phoma* Coefficient Estimates at 17% mg/mg (SE), *N* = 315
Control	NA	NA	3.3 (0.2)*	7.5 (0.2)*	5.2 (0.1)*
*Anacardium excelsum*	Immature	Seed	−3.3 (0.3) A*	−6.0 (0.5) A*	2.0 (0.4) AC*
		Pericarp	−6.9 (0.6) B*	−6.1 (0.5) A*	**−**0.4 (0.4) B
	Mature	Seed	−2.2 (0.3) C*	−6.0 (0.5) A*	1.2 (0.4) A*
		Pericarp	−23.9 (NA) *^a^	−7.6 (0.5) B*	−1.0 (0.4) B*
	Young	Leaf	−8.3 (1.1) B*	−3.6 (0.5) C*	2.5 (0.4) C*
*Antirhea tricantha*	Immature	Fruit	−2.3 (0.2) A*	−1.8 (0.5) A*	4.9 (0.4) A*
	Mature	Seed	−2.0 (0.2) A*	−0.6 (0.5) A	3.1 (0.4) B*
		Pericarp	−23.6 (NA) *^a^	0.7 (0.5) B	1.8 (0.4) C*
*Bonamia tricantha*	Immature	Diaspore	−5.8 (0.5) A*	−0.6 (0.5) A	1.8 (0.4) AC*
		Capsule	−3.8 (0.4) B*	−2.5 (0.5) B*	1.7 (0.4) AC*
	Mature	Diaspore	−6.9 (0.5) A*	0.4 (0.5) A	3.8 (0.4) B*
		Capsule	−2.1 (0.3) C*	−2.2 (0.5) B*	0.7 (0.4) A
	Young	Leaf	−5.7 (0.4)A*	−2.7 (0.5) B*	2.1 (0.4) C*
*Castilla elastica*	Immature	Seed	−2.3 (0.4) A*	1.0 (0.5) A*	5.3 (0.4) A*
		Pericarp	−0.3 (0.8) B	0.9 (0.5) A*	3.1 (0.4) B*
	Mature	Seed	−5.6 (0.3) C*	1.3 (0.5) A*	5.8 (0.4) A*
		Pericarp	−4.9 (0.3) D*	−0.8 (0.5) B	2.6 (0.4) B*
		Calyx	−2.4 (0.2) E*	0.8 (0.5) A	6.2 (0.4) A*
*Cecropia longipipes*	Immature	Fruit	−0.3 (0.5) A	1.4 (0.5) A*	6.5 (0.4) A*
	Mature	Diaspore	−6.2 (0.4) B*	2.3 (0.5) A*	3.7 (0.4) B*
		Pulp	−0.1 (0.6) A	0.2 (0.5) B	2.9 (0.4) B*
	Young	Leaf	−0.3 (0.4) A	1.3 (0.5) AB*	5.2 (0.4) D*
*Cecropia peltata*	Immature	Fruit	−1.0 (0.4) A*	−1.0 (0.5) A*	5.5 (0.4) A*
	Mature	Diaspore	−7.8 (0.5) B*	0.6 (0.5) B	0.2 (0.4) B
		Pulp	−9.2 (1.0) B*	1.1 (0.5) B*	1.3 (0.4) B*
*Jacaranda copaia*	Immature	Fruit	−0.8 (0.5) A	−2.2 (0.5) A*	2.9 (0.4) AB*
	Mature	Diaspore	−3.7 (0.4) B*	−1.8 (0.5) A*	3.4 (0.4) A*
		Capsule	−3.9 (0.4) B*	−2.0 (0.5) A*	2.2 (0.4) B*
*Luehea seemannii*	Immature	Diaspore	−3.0 (0.3) A*	−3.0 (0.5) A*	3.2 (0.4) A*
		Capsule	−1.2 (0.3) B*	−3.3 (0.5) A*	3.7 (0.4) A*
	Mature	Diaspore	−4.5 (0.3) C*	1.4 (0.5) B*	6.8 (0.4) B*
		Capsule	−2.5 (0.3) D*	−1.2 (0.5) C*	4.9 (0.4) C*
*Nectandra umbrosa*	Immature	Seed	−8.1 (1.0) A*	−3.8 (0.5) A*	1.7 (0.4) A*
		Pericarp	−8.1 (1.0) A*	−4.4 (0.5) A*	2.5 (0.4) A*
	Mature	Seed	−23.1 (NA)*^a^	−8.2 (0.5) B*	−2.2 (0.4) B*
		Pericarp	−4.3 (0.4) B*	−2.5 (0.5) C*	4.1 (0.4) C*
*Stigmaphyllon hypargyreum*	Immature	Seed	−1.2 (0.3) A*	−2.1 (0.5) AB*	2.2 (0.4) A*
		Pericarp	−2.5 (0.3) B*	−2.5 (0.5) A*	4.3 (0.4) B*
	Mature	Seed	−2.4 (0.3) B*	−1.9 (0.5) AB*	4.3 (0.4) C*
		Pericarp	−2.8 (0.2) B*	−0.3 **(0.5)** C	5.9 (0.4) D*
	Young	Leaf	−2.5 (0.4) B*	−1.2 (0.05) B*	0.85 (0.4) E*
*Tapirira guianensis*	Immature	Seed	−0.6 (0.4) A	−5.4 (0.5) A*	2.0 (0.4) A*
		Pericarp	16.4 (NA)^b^	−5.8 (0.5) A*	0.7 (0.4) B
	Mature	Seed	−6.5 (0.6) B*	0.0 (0.5) B	4.3 (0.4) C*
		Pericarp	−3.5 (0.4) C*	−2.2 (0.5) C*	3.9 (0.4) C*
	Young	Leaf	−0.4 (0.4) A	−2.5 (0.5) C*	2.5 (0.4) A*

*Notes:* The coefficient estimates and standard errors from generalized linear mixed models of the proportion of surviving *Artemia* and fungal hyphal growth on extracts compared to negative controls. Coefficient estimates are differences in hyphal growth (mm) in plant extracts from negative controls for fungal bioassays, and the log of the odds ratio between plant extracts and negative controls for the *Artemia* bioassay. Stars indicate significant differences of responses of organisms in treatments compared to controls at the 0.05 significance level. Letters indicate differences among means within species. ^a^All shrimp died or ^b^ survived and standard errors were not estimated..

**Table 7:** Number of species that significantly inhibited bioassay organisms compared to controls within each dispersal mode.

		Number of Toxic Species					
Fruit Development Stage	Bioassay	Vertebrate-dispersed			Wind-dispersed		
		Whole Fruit	Seed	Pericarp	Whole Fruit	Seed	Pericarp
**A. Toxicity of Immature fruit**	***n***	**3**	**4**	**4**	**1**	**3**	**3**
	*Artemia*	2	3	2	0	3	3
	*Fusarium*	2	3	3	1	2	3
	*Phoma*	0	0	0	0	0	0
**B. Toxicity of Mature Fruit**	***n***	−	**7**	**7**	−	**4**	**4**
	*Artemia*	−	7	6	−	4	4
	*Fusarium*	−	2	3	−	2	3
	*Phoma*	−	1	1	−	0	0

*Notes:* Seed refers to extracts from the diaspore or seed, and pericarp refers to extracts from the pericarp, capsule, or pulp. If an extract kills all shrimp, it is counted as significantly more toxic, and likewise, if zero shrimp died, it is counted as significantly less toxic.

**Table 8:** Concentrations at which 50% inhibition occurs (ED_50_).

Species	Stage	Fruit Part	% extract (mg)/fruit part (mg)*	ED_50_		
				*Artemia* mg/ml (SE)	*Fusarium sp.* % mg/mg (SE)	*Phoma* % mg/mg (SE)
*Anacardium excelsum*	Immature	Seed	26	0.57 (0.085)	6.7 (0.6)	−
		Pericarp	45	0.027 (0.0022)	4.6 (0.8)	−
	Mature	Seed	40	29 (18)†	10.6 (0.9)	−
		Pericarp	34	0.023 (0.0026)	4.4 (0.4)	0.14 (0.02)
	Young	Leaf	−	0.11 (0.010)	23.1 (7.9)†	−
*Antirhea trichanta*	Immature	Fruit	14	−	19.5 (3.5)†	−
	Mature	Seed	27	−	18.8 (4.6)†	−
		Pericarp	57	0.021 (0.0023)	−	−
*Bonamia tricantha*	Immature	Diaspore	25	0.033 (0.0031)	23.8 (16)†	−
		Capsule	15	1.2 (0.35)†	71.6 (48.4)†	−
	Mature	Diaspore	14	0.091 (0.0096)	−	−
		Capsule	5	6.4 (3.3)†	83.8 (49.4)†	−
	Young	Leaf	−	0.29 (0.031)	−	−
*Castilla elastica*	Immature	Seed	12	23 (20)†	−	−
		Pericarp	11	−	−	−
	Mature	Seed	12	0.31 (0.027)	−	−
		Pericarp	87	0.48 (0.045)	30.6 (12.3)†	−
		Calyx	50	23 (18)†	−	−
*Cecropia longipes*	Immature	Fruit	14	−	−	−
	Mature	Diaspore	15	0.22 (0.02)	−	−
		Pulp	−	4.4 (0.76)†	−	−
	Young	Leaf	−	225 (215)†	−	−
*Cecropia peltata*	Immature	Fruit	21	5728 (7705)†	24.3 (7.0)†	−
	Mature	Diaspore	26	0.22 (0.020)	−	−
		Pulp	79	0.22 (0.020)	−	−
*Jacaranda copaia*	Immature	Fruit	55	−	27.2 (6.3) †	−
	Mature	Diaspore	11	0.75 (0.093)	39.5 (22.0)†	−
		Capsule	16	0.61 (0.088)	23.4 (3.9) †	−
*Leuhea seemannii*	Immature	Diaspore	10	3.4 (1.5)	18.4 (1.3) †	−
		Capsule	14	NA	18.4 (2.9) †	−
	Mature	Diaspore	12	0.53 (0.064)	−	−
		Capsule	2	21.4 (12.2) †	37.9 (23.4)†	−
*Nectandra umbrosa*	Immature	Seed	−	0.26 (0.026)	16.1 (1.7)	−
		Pericarp	54	0.20 (0.020)	11.3 (2.1)	−
	Mature	Seed	6	0.034 (0.0047)	0.28 (0.10)	0.21 (0.05)
		Pericarp	36	0.48 (0.079)	−	−
*Stigmaphyllon hypargyreum*	Immature	Seed	29	22.1 (19.4) †	17.5 (1.5) †	−
		Pericarp	21	1.08 (0.296) †	20.1 (2.2) †	−
	Mature	Seed	33	3.05 (1.14) †	38.6 (14.8)†	−
		Pericarp	4	1.28 (0.25) †	−	−
	Young	Leaf	−	7.01 (4.05) †	66.5 (54.2)†	−
*Tapirira guianensis*	Immature	Seed	80	−	9.9 (0.5)	−
		Pericarp	63	−	9.5(0.7)	−
	Mature	Seed	66	0.250 (0.029)	NA	−
		Pericarp	40	1.07 (0.23) †	34.4 (13.5)†	−
	Young	Leaf	−	114 (99.8)†	39.1 (19.5)†	−
Positive Control	−	−	−	0.21 (0.02) ul/ml	353 (34) mg/ml	387 (12) mg/ml

* Percent extract/fruit part is the estimated concentration of the extract (dry mass, g) per fruit part (dry mass, g) from which it was extracted.

† Estimated values are higher than concentrations tested.

### Is the Seed or Pericarp of Mature Fruit more Toxic?

**Figure 4:**
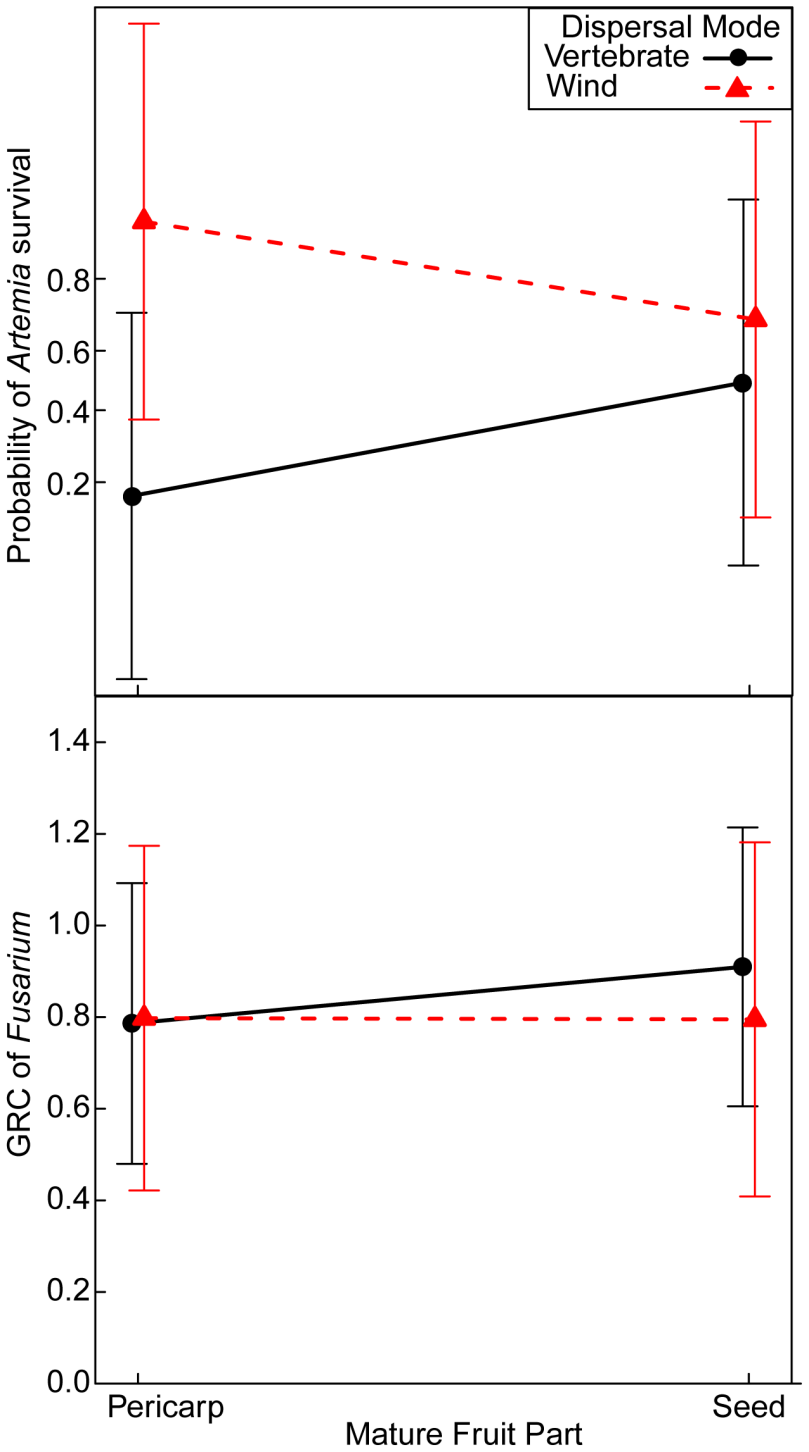
Is the seed or pericarp of mature fruit more toxic? Shown is the interaction between fruit part and dispersal mode from generalized linear mixed models explaining the proportion of surviving *Artemia franciscana* and hyphal growth of *Fusarium* relative to negative controls in mature fruit extracts.

Contrary to my prediction that the mature pericarp is less toxic than the seed of vertebrate-dispersed species (1a), the mature pericarp was more toxic than the seed for vertebrate-dispersed species in both *Artemia* and *Fusarium* bioassays and in one case in the *Phoma* bioassay. Comparing a suite of nested models, the model with the lowest AIC values for both bioassays included fruit part (Table 2), suggesting a difference between mature seed and pericarp toxicity. For *Artemia*, the full model with dispersal mode, fruit part, and their interaction had the lowest AIC value (Table S1A); for *Fusarium*, three models had AIC values within two AIC units of the best-fit model, and the best-fit model included fruit part only. The mature pericarp from vertebrate-dispersed species was more toxic to *Artemia* than the seed, and the mature seed of wind-dispersed species was more toxic than the pericarp (Table S1A, Fig. 4). The mature pericarp was more toxic to *Fusarium* than the seed across dispersal modes in the best-fit model (CE (SE) = −0.12 (0.04)), with the mature pericarp of vertebrate-dispersed species being slightly more toxic than seeds in the second best-fit model (ΔAIC = 1.9, Fig. 4), which included an interaction between fruit part and dispersal mode (Table S1B). Although I did not use a model comparison approach in the *Phoma* bioassay, two vertebrate-dispersed species had one toxic mature fruit extract. The seed was more toxic to *Phoma* than the pericarp of *Nectandra umbrosa*, and the pericarp was more toxic than the seed of *Anacardium excelsum* (Fig. 2). Activity of mature seed among species increased significantly with activity of mature pericarp in the *Fusarium* bioassay (*t_9_* = 2.4, *r = *0.63, *P*<0.05; Table 9B). Activity of mature seed and pericarp among species was not correlated in the *Artemia* (Table 9A) and *Phoma* bioassays (Table 9C).

**Table 9:** Correlations between plant parts within bioassays.

Bioassay	Fruit Part 1	Fruit Part 2	*t*	*df*	*r*	*P*
**A. ** ***Artemia***	Immature Seed	Immature Pericarp	1.97	5	0.66	0.1064
	Mature Seed	Mature Pericarp	−0.98	9	−0.31	0.3547
	Immature Pericarp	Mature Pericarp	0.88	9	0.28	0.4041
	**Immature Seed**	**Mature Seed**	**2.99**	**9**	**0.71**	**0.0152**
	Immature Pericarp	Leaf	1.38	2	0.70	0.3012
	Immature Seed	Leaf	1.78	2	0.78	0.2166
	Mature Pericarp	Leaf	2.15	3	0.78	0.1205
	Mature Seed	Leaf	−0.95	3	−0.48	0.4106
**B. ** ***Fusarium***	**Immature Seed**	**Immature Pericarp**	**9.05**	**5**	**0.97**	**0.00023**
	**Mature Seed**	**Mature Pericarp**	**2.43**	**9**	**0.63**	**0.0379**
	**Immature Pericarp**	**Mature Pericarp**	**2.97**	**9**	**0.70**	**0.0157**
	**Immature Seed**	**Mature Seed**	**2.51**	**9**	**0.64**	**0.0331**
	**Immature Pericarp**	**Leaf**	**3.55**	**3**	**0.90**	**0.0380**
	Immature Seed	Leaf	2.21	3	0.79	0.1141
	Mature Pericarp	Leaf	2.13	3	0.78	0.1232
	Mature Seed	Leaf	1.66	3	0.69	0.1941
**C. ** ***Phoma***	Immature Seed	Immature Pericarp	0.96	5	0.39	0.3822
	Mature Seed	Mature Pericarp	0.98	9	0.31	0.3541
	Immature Pericarp	Mature Pericarp	1.03	9	0.32	0.3317
	Immature Seed	Mature Seed	0.51	9	0.167	0.6238
	Immature Pericarp	Leaf	0.91	3	0.46	0.4314
	**Immature Seed**	**Leaf**	**3.38**	**3**	**0.89**	**0.0431**
	Mature Pericarp	Leaf	−0.36	3	−0.20	0.7411
	Mature Seed	Leaf	−0.20	3	−0.11	0.857

### During which Development Stage is Fruit Most Toxic?

Contrary to my prediction that mature fruit is less toxic than immature fruit (1b), mature fruit parts of vertebrate-dispersed species were more toxic than immature fruit parts in the *Artemia* bioassay and for one plant species in the *Phoma* bioassay. Contrary to my prediction that the mature pericarp decreases more in toxicity across developmental stages compared to the seed (1b), the toxicity of the pericarp did not decrease from the immature to the mature stage more than the seed of vertebrate-dispersed fruits in the *Aretmia* and *Fusarium* bioassays. Comparing a suite of nested models, the model with the lowest AIC values for both bioassays included fruit developmental stage (Table 3), suggesting a difference between immature and mature fruits. For *Artemia*, the full model including dispersal mode, fruit developmental stage, fruit part, and their interactions had the lowest AIC value (Table S2A). For *Fusarium*, two models had AIC values within two AIC units of the best-fit model. The best-fit model included dispersal mode, fruit part, and their interaction plus fruit developmental stage (Table S2B). However, the simplest model included these same terms but did not include fruit developmental stage (ΔAIC = 0.2), suggesting less support for differences in fruit toxicity to *Fusarium* between immature and mature stages.

**Figure 5:**
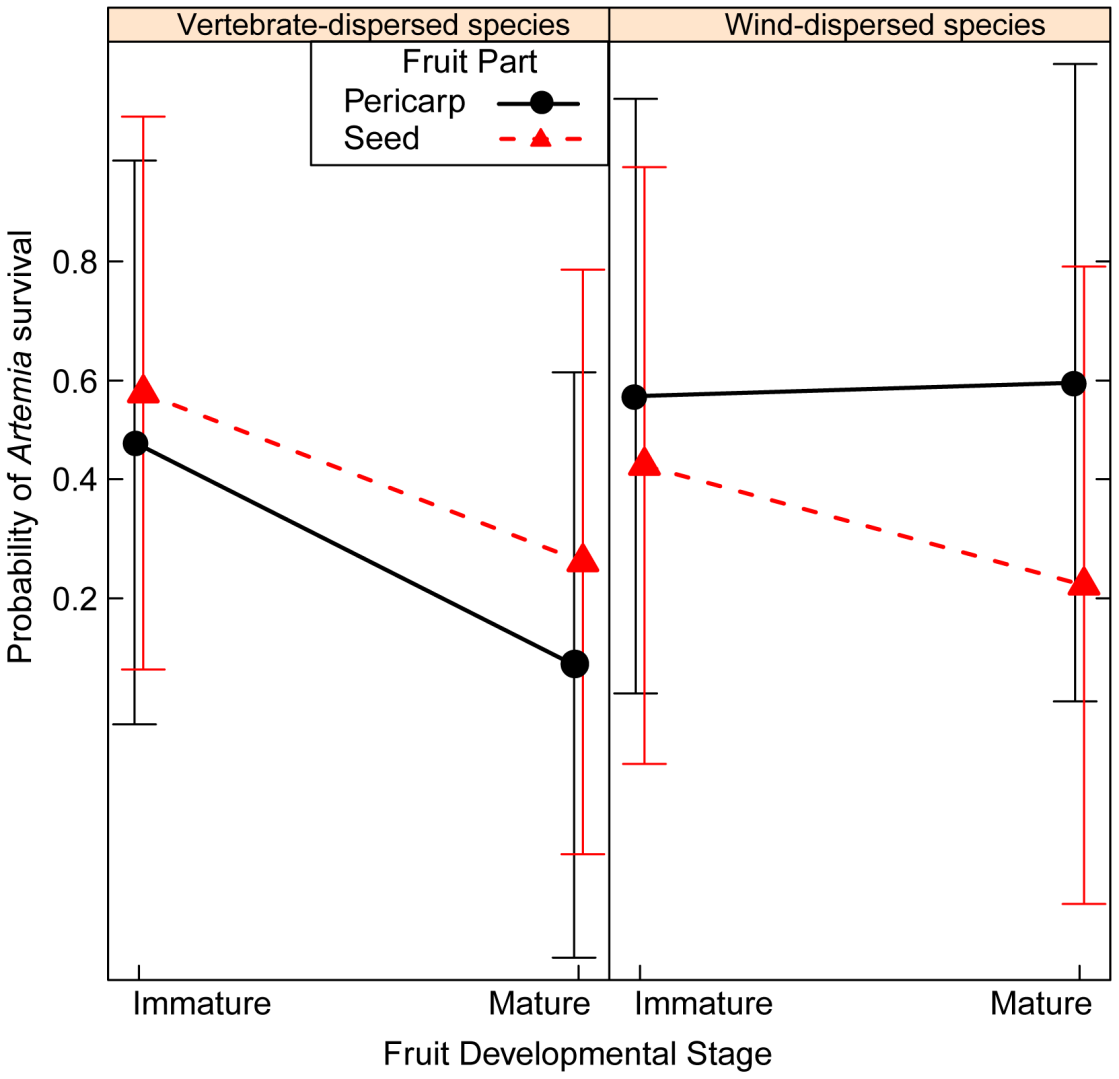
During which developmental stage are fruits most toxic to *Artemia?* Shown is the interaction among fruit developmental stage, fruit part, and dispersal mode from a generalized linear mixed model of the proportion of surviving *Artemia franciscana* for a subset of seven species.

**Figure 6:**
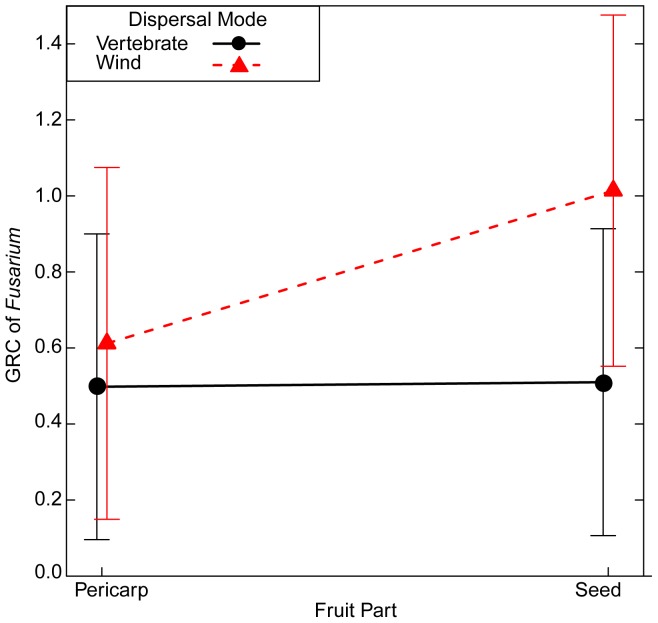
During which developmental stage are fruits most toxic to *Fusarium?* Shown is the interaction between fruit part and dispersal mode from a generalized linear mixed model of the hyphal growth of *Fusarium* relative to negative controls (GRC) for a subset of seven species.

In the *Artemia* bioassay, mature fruit of seed and pericarp were more toxic than immature fruit parts of vertebrate-dispersed species (Fig. 5). Similarly for wind-dispersed species, the mature seed of wind-dispersed species was slightly more toxic than the immature seed, and the mature and immature pericarp had equivalent toxicity to *Artemia* (Fig. 5). In the best-fit model of *Fusarium*, mature fruit extracts slightly increased GRC of *Fusarium* relative to immature fruits (CE (SE) = 0.17 (0.12)). In the *Phoma* bioassay, I did not use a model comparison approach, but there were no toxic immature extracts, and the mature seed was more toxic than the immature seed of *Nectandra umbrosa* (Fig. 2). For both immature and mature stages of fruit, the pericarp of vertebrate-dispersed species was slightly more toxic than the seeds in the *Artemia* bioassay (Fig. 5) and had equivalent toxicity to seeds in the *Fusarium* bioassay (Fig. 6). In the *Phoma* bioassay, the toxicity of the pericarp was equivalent between developmental stages for *Anacardium excelsum* (Fig. 2). For wind-dispersed species, the seed was more toxic to *Artemia* than the pericarp (Fig. 5), while the pericarp was slightly more toxic to *Fusarium* than the seed across developmental stages (Fig. 6).

Activity of immature seeds (including extracts from the entire fruit) significantly increased with activity of mature seeds among species in *Artemia* (*t_9_* = 3.0, *r = *0.71, *P*<0.05) and *Fusarium* (*t_9_* = 2.5, *r = *0.64, *P*<0.05) bioassays. The activity of immature pericarp (including extracts from the entire fruit) was not significantly correlated with the mature pericarp or the immature seed for the *Artemia* bioassay (Table 9A), but significantly increased with mature pericarp (*t_9_* = 3.0, *r = *0.70, *P*<0.05) and the immature seed for *Fusarium* bioassays (*t_5_* = 9.1, *r = *0.97, *P*<0.001; Table 9B). For the *Phoma* bioassay, the activity of immature seed and pericarp was not correlated nor was the activity of immature fruit parts (including extracts from the entire fruit) with mature fruit parts among plant species (Table 9C).

### Are Fruit or Young Leaves more Toxic?

Fruits were not consistently more toxic than leaves (1c). For *Artemia* and *Fusarium,* the best-fit models did not include fixed effects of dispersal mode or plant part (Table 4), suggesting no overall pattern of leaf versus fruit toxicity for these five plant species. For both bioassays, there were three models that had AIC values within two AIC units of the best-fit model. There was some support for slightly higher *Artemia* survivorship (ΔAIC = 1.5, CE (SE) = 0.49 (1.69)) and GRC of *Fusarium* (ΔAIC = 1.3, CE (SE) = 0.23 (0.35)) in extracts from wind-dispersed species compared to vertebrate-dispersed species. There was also some support for higher *Artemia* survivorship on fruit compared to leaf extracts ((ΔAIC = 1.9, CE (SE) = 1.94 (2.64)), and lower GRC of *Fusarium* on fruit compared to leaf extracts (ΔAIC = 1.8, CE (SE) = −0.08 (0.16)).

Leaf extracts of three species were toxic to *Artemia*, four to *Fusarium,* and none to *Phoma* (Table 6). For *Anacardium excelsum*, the leaf extract had similar toxicity to *Artemia* as the immature pericarp, lower toxicity than the mature pericarp, and higher toxicity than immature and mature seed extracts (Fig. 2). *A. excelsum* leaf extract had lower toxicity than all fruit extracts to *Fusarium* and was not toxic to *Phoma*, whereas the mature pericarp was toxic to *Phoma*. In all bioassays, the *Cecropia longipes* leaf extract was not toxic, whereas the seed extract was active against *Artemia*. *Tapirira guianensis* leaf extract was not toxic to *Artemia*, although the mature fruit parts were toxic. *T. guianensis* leaf extract had similar toxicity to *Fusarium* as the mature pericarp and lower toxicity than immature fruit extracts. For *Bonamia trichantha*, leaf extracts had similar toxicity to *Artemia* as seed extracts and higher toxicity than pericarp extracts (Fig. 3). *B. trichantha* leaf extracts were similarly toxic to *Fusarium* as pericarp extracts. *Stigmaphyllon hypargyreum* leaf extracts had higher toxicity than the immature seed extract and similar toxicity as all other extracts to *Artemia*. It had higher toxicity than the mature pericarp, lower toxicity than immature pericarp, and similar toxicity as immature and mature seed extracts to *Fusarium.* Activity of leaf extracts across five species was not correlated with activity of fruit parts at either the immature or mature stage for the *Artemia* bioassay (Table 9A). Activity of the leaf extract was positively correlated with the immature pericarp for *Fusarium* (*t_3_* = 3.6, *r = *0.90, *P*<0.05; Table 9B) and the immature seed for *Phoma* (*t_3_* = 3.4, *r = *0.89, *P*<0.05; Table 9C).

### Is Fruit Toxicity Related to Morphology?

**Figure 7:**
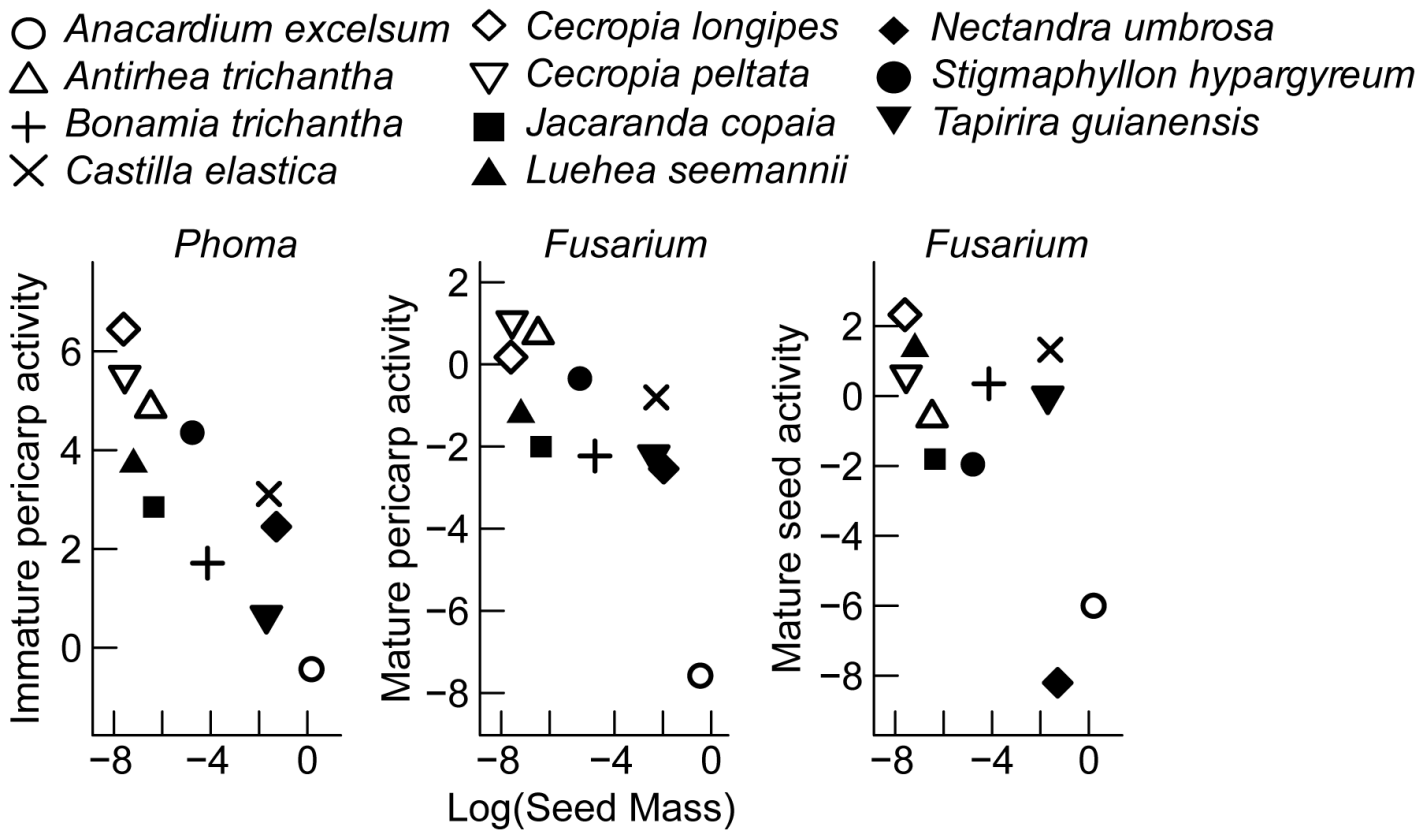
Correlation between seed size and fruit toxicity for eleven tree and vine species.

Toxicity increased with seed size in the *Fusarium* bioassay and no other bioassay, and toxicity was not correlated with physical protection contrary to my prediction that toxicity would be either positively or negatively correlated (2). Log seed reserve dry mass was negatively correlated with the activity of mature seed (*t_9_* = −2.3, *P*<0.05, *r* = −0.62) and mature pericarp (*t_9_* = −3.3, *P*<0.01, *r* = −0.74) against *Fusarium* (Table 10B). Mature fruit extracts from heavier seeds were more toxic against *Fusarium* compared to lighter seeds (Fig. 7). Log seed reserve dry mass was negatively correlated with the beneficial activity of immature pericarp (including whole fruits) to *Phoma* (*t_9_* = −4.4, *P*<0.01, *r* = −0.82; Table 10C). Immature pericarp from fruit with heavier seeds had lower positive effects on hyphal growth of *Phoma* compared to lighter seeds (Fig. 7). No other extracts had effects that were significantly correlated with log of seed size or physical defense per diaspore (Table 10).

**Table 10:** Correlations between fruit morphology and extract activity of fruit parts within bioassays.

Bioassay	Fruit Morphology	Fruit Extract	*t*	*df*	*r*	*P*
**A. ** ***Artemia***	Log (seed mass)	Immature seed	−1.46	9	−0.44	0.1775
	Log (seed mass)	Immature pericarp	−0.02	9	−0.006	0.9849
	Log (seed mass)	Mature Seed	−0.86	9	−0.27	0.4135
	Log (seed mass)	Mature Pericarp	−0.71	9	−0.23	0.4958
	Physical defense	Immature seed	1.57	9	0.46	0.1501
	Physical defense	Immature pericarp	0.18	9	0.06	0.8626
	Physical defense	Mature Seed	2.13	9	0.58	0.0615
	Physical defense	Mature Pericarp	−0.82	9	−0.26	0.4357
**B. ** ***Fusarium***	Log (seed mass)	Immature seed	−1.88	9	−0.53	0.0928
	Log (seed mass)	Immature pericarp	−2.06	9	−0.57	0.0699
	**Log (seed mass)**	**Mature Seed**	**−2.34**	**9**	**−0.62**	**0.0439**
	**Log (seed mass)**	**Mature Pericarp**	**−3.30**	**9**	**−0.74**	**0.0093**
	Physical defense	Immature seed	0.25	9	0.08	0.8097
	Physical defense	Immature pericarp	0.36	9	0.12	0.725
	Physical defense	Mature Seed	1.03	9	0.32	0.3312
	Physical defense	Mature Pericarp	1.35	9	0.41	0.2096
**C. ** ***Phoma***	Log (seed mass)	Immature seed	−2.03	9	−0.56	0.0731
	**Log (seed mass)**	**Immature pericarp**	**−4.36**	**9**	**−0.82**	**0.0018**
	Log (seed mass)	Mature Seed	−0.78	9	−0.25	0.4551
	Log (seed mass)	Mature Pericarp	−0.61	9	−0.2	0.5563
	Physical defense	Immature seed	0.57	9	0.19	0.5808
	Physical defense	Immature pericarp	1.46	9	0.44	0.1772
	Physical defense	Mature Seed	0.64	9	0.21	0.5371
	Physical defense	Mature Pericarp	−0.01	9	−0.005	0.9896

### Does Fruit Toxicity Help Explain Variation in Fruit Development and Seed Germination?

**Figure 8:**
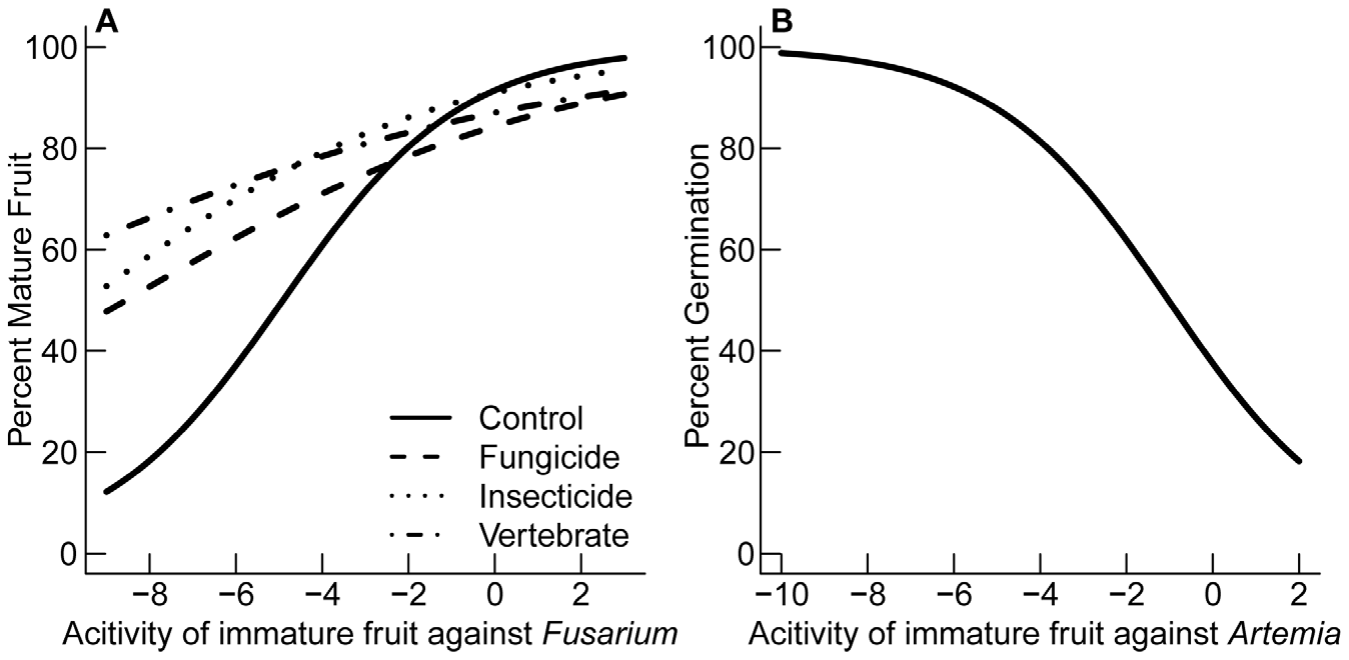
The effect of natural enemy removal treatments and fruit toxicity on seed viability. (**a**) The probability of fruit maturation and (**b**) seed germination of tropical trees and vines in central Panama. Lines are best fits of generalized linear mixed models (Tables S3–S4).

The prediction that toxicity would increase plant survivorship (3) was supported at the germination stage but not the fruit development stage. Comparing a suite of nested models, the models with the lowest AIC values for fruit development and germination included at least one measure of toxicity to either *Artemia* or *Fusarium* (Table 11), suggesting fruit toxicity is important in explaining variation in plant survivorship. After accounting for differences in fruit morphology, the best-fit model explaining variation in fruit development included immature fruit toxicity to *Artemia* and the interaction of natural enemy removal treatments and immature fruit toxicity to *Fusarium* (Table S3), and the second best-fit model included immature fruit toxicity to *Fusarium* (ΔAIC = 1.9, Table 11). The best-fit model explaining variation in germination after accounting for differences in fruit morphology, included toxicity of immature fruit to *Artemia* (Table S4), and the second best-fit model included both toxicity of immature fruit to *Artemia* and *Fusarium* (ΔAIC = 1.3, Table 11). The proportion of fruit that reached maturity increased with reduced toxicity of immature extracts against *Fusarium* (as indicated by increased hyphal growth of *Fusarium*), with lower fruit development in controls compared to treatments of fruit with higher toxicity (Fig. 8A). Germination increased with toxicity to *Artemia* (as reflected in decreased *Artemia* survival in immature extracts; Fig. 8B).

**Table 11:** Does fruit toxicity help explain variation in fruit development and seed survival?

	AIC	
Terms in Model	Fruit development	Germination
Natural enemy removal treatment×Toxicity to *Artemia*+Natural enemy removal treatment×Toxicity to *Fusarium*	7772.8	6863.7
Toxicity to *Artemia*+Natural enemy removal treatment×Toxicity to *Fusarium*	**7767.9**	6859.7
Natural enemy removal treatment×Toxicity to *Artemia*+Toxicity to *Fusarium*	7774.3	6859.5
Toxicity to *Artemia*+Toxicity to *Fusarium*	7771.5	**6855.7**
Toxicity to *Artemia*	7773.0	**6854.4**
Toxicity to *Fusarium*	**7769.9**	6858.7
No toxicity	7772.9	6857.9

*Notes:* Comparison of AIC values for generalized linear mixed effects models of fruit development and germination. AIC values of models within two AIC of best-fit models are in bold.

## Discussion

Every species from the individuals included in this study was toxic to a bioassay organism in either its seed or pericarp at some point during fruit development, and the pattern of chemical defense depended on dispersal mode and bioassay organism. Mature fruit of vertebrate-dispersed species was just as toxic or more toxic than immature fruit, and the toxicity of ripe pulp did not decline with developmental stages. Because there were several cases in which mature fruit were more toxic than leaves, the results suggest that chemical defense in ripe fruit may not be constrained by leaf toxicity. As toxicity of immature fruit to bioassay organisms partly explained interspecific variation in predispersal seed survival, chemical defenses in fruit should be considered an important mechanism in mediating interactions with generalist natural enemies.

### Patterns of Fruit Toxicity

Variation of fruit toxicity within bioassays depended on fruit parts, developmental stages, and dispersal mode. Contrary to my prediction, the mature pericarp of vertebrate-dispersed species was just as toxic or more toxic than the seed for *Artemia*, *Fusarium* (Fig. 4), and in one case for *Phoma* (Fig. 2), and the toxicity of pericarp of vertebrate-dispersed species did not decline with developmental stages compared to seeds (Fig. 5, 6). Secondary metabolites in ripe fruit pulp may play an important role in slowing fungal growth [7], [41], deterring frugivores [49], and influencing gut retention time [13]. Although I predicted mature fruit of vertebrate-dispersed species to be less toxic than immature fruit, mature fruit parts were more toxic against *Artemia* than immature fruit (Fig. 5), and mature seed of one vertebrate-dispersed species was more toxic than immature seeds against *Phoma* (Fig. 2). In wind-dispersed species, mature seed was also more toxic than immature seed against *Artemia* (Fig. 5). During fruit development, immature fruits were slightly more toxic than the mature fruits against *Fusarium* independent of dispersal mode, but variation in toxicity may be better explained by dispersal mode and fruit part, as developmental stage was not included in the second-best model. Higher toxicity at the mature fruit developmental stage may reflect higher value of fully mature fruit and/or higher predation pressures at this stage. Chemical defenses in mature seeds have been shown to reduce fungal growth and *Artemia* survivorship; this may correspond with increased persistence in the soil seed bank [30].

Bioassays are an effective method to determine toxicity of a range of organisms to the entire suite of compounds within a plant, providing information on the potential responses from generalist natural enemies. In this study, there were differences among the bioassay organisms, and toxicities of fruit extracts between bioassay organisms were significantly correlated for only three of the fifteen combinations of plant extract and bioassay organism (Table S5). Among the fungi, *Fusarium* was more sensitive to negative effects of plant extracts than *Phoma.* The two fungi belong to different classes of Ascomycota, *Phoma* to Dothideomycetes and *Fusarium* Sordariomycetes; differences in their genetic structure and therefore enzymatic production could result in their different responses to plant toxins (*e.g.* detoxicification or active secretion of the toxin) [50], [51].

### Fruit and Leaf Toxicity

Overall, activity of extracts did not depend on plant part (leaf or fruit) or dispersal mode (vertebrate or wind), and toxicity between fruit and leaves was not correlated among species, except for the toxicity of leaves and the immature pericarp to *Fusarium*. There was some support for higher toxicity of plant extracts from vertebrate-dispersed species compared to wind-dispersed species, and there was some support for higher fruit toxicity compared to leaf toxicity to *Fusarium* and higher leaf toxicity compared to fruit toxicity to *Artemia*. However, there were very few species in this study and more research needs to be conducted to determine the generality of these results.

Although leaf extracts were as toxic or more toxic than some fruit extracts, there were several instances in which immature or mature fruit extracts were more toxic than leaves. Three of the five species had at least one mature fruit part with higher toxicity than young leaves in at least one bioassay, and three had at least one immature fruit part with higher toxicity than young leaves (Fig. 2,3; Table 6), suggesting increased allocation to defense of either mature and immature fruit during development for these species. These results may lend support to the hypothesis that fruit toxicity is not constrained by chemical defense in green tissue [12]. Defense within a plant is expected to be allocated to tissue in direct proportion to the risk of predation and its fitness value and in inverse proportion to the cost of defense [52]. Future studies should quantify allocation of chemical defense within the plant and compare these to risk of consumption by herbivores, seed predators, and pathogens throughout fruit development and seedling establishment. For ripe pulp of fleshy fruits, interactions with seed dispersers will also have to be considered as secondary metabolites may have multiple functions including attraction [4].

### Fruit Morphology and Toxicity

In this study, activity against *Artemia* was unrelated to seed size, but seed size increased with toxicity of mature fruit parts against *Fusarium* and positive hyphal growth of *Phoma* on immature pericarp extracts compared to controls. This suggests species with larger seed reserves for seedling establishment invest more in chemical defense for successfully developed seeds than species with smaller seeds. However, future studies should be conducted to determine whether this pattern holds for a greater number of individuals and species. How other factors influence chemical defenses, such as seed longevity and natural enemy pressures prior to germination and seedling establishment, should also be further investigated. The three larger-seeded species for which data are available (*i.e.*, *Anacardium excelsum, Castilla elastica*, and *Tapirira guianensis*) have faster germination rates and are viable for shorter time periods than the smaller-seeded species included in this study [53]. The smaller-seeded species include species that have seed banks, and may therefore be attacked by natural enemies over longer periods of time than the larger-seeded species. Veldman *et al.* found that for several small-seeded Neotropical species, seed bank longevity was associated with greater seed toxicity [30]. In the tropical forest plant communities studied here, larger seeds may have greater natural enemy pressure over shorter periods of time whereas smaller seeds may defend themselves for longer periods of time.

The proportion of a diaspore allocated to physical defense did not correlate either positively or negatively with toxicity. A negative correlation might be expected given limited resources for defense. This may be due to total energy constraints, or be related to the particular biosynthetic pathways involved – for example, in chili fruits, capsaicin and lignin, which contribute to chemical and physical defenses, respectively, may compete for the same molecular precursors, and therefore the production of one may limit the production of the other [13]. On the other hand, a positive correlation would have suggested that plant species differ mainly in their overall allocation to defense, with species that allocate more to physical defenses also allocating more to chemical defenses. The lack of any relationship between physical defenses and toxicity suggests that species vary considerably both in total resource allocation to defense and in their relative allocation to physical and chemical defense.

### Seed Survival and Toxicity

Fruit development increased with hyphal growth of fungi on immature extracts compared to controls, and germination increased with toxicity to *Artemia*. The second result suggests that increased toxicity to *Artemia* indicates better defenses against generalist insect seed predators. Two nonexclusive hypotheses may explain why seed survival increased with reduced toxicity to fungi. It may be the case that species with higher toxicity to fungi have greater overall pressure from fungal pathogens compared to less toxic species, as is indicated by the higher fruit development in natural enemy removal treatments compared to controls for species with higher toxicity. The species with the highest toxicity to *Fusarium*, *Anacardium excelsum,* also had the highest incidence of fungal infection [26]. Alternatively, species with extracts that increased fungal hyphal growth may have mutualistic relationships with fungi. Potentially, smaller-seeded species have mutualistic interactions with fungi as the immature pericarp from these species tended to increase fungal hyphal growth of *Phoma* compared to larger-seeded species. Endophytic fungi may offer benefits to seed survival during fruit development by reducing the colonization and growth of pathogenic fungi [54].

### Generalist vs. Specialist Natural Enemies

The responses of the bioassay organisms used in this study are expected to be good indicators of potential responses of generalist natural enemies. Their relevance to the potential responses of specialist enemies is more tenuous. Fruits and seeds are consumed by both generalist and specialist natural enemies, and both groups are expected to influence the evolution of plant defenses [52]. Insect seed predators tend to be specialized on one or a few related plant species [29], [55], [56], while vertebrates tend to be generalists [57]. While less is known for the host range of pathogens, especially in tropical forests, several studies demonstrate that many foliar and soil fungal pathogen strains may have a limited host range [39], [58]. At the same time, other studies suggest that soil pathogens that are responsible for a large fraction of seed and seedling mortality have an intermediate level of specificity [59]. Thus, an important open question is the impact of fruit toxicity on more specialized enemies.

### Caveats

The results of this study describe the variation in plant toxicity for the community of fruiting trees accessible from each crane. Because of the high diversity of plants and limited range of the canopy arm, this study included small numbers of individuals for the eleven fruiting species accessible from the canopy crane. Whether these individuals are representative of each species depends on the variation in fruit toxicity within species. In an experiment examining the effect of resource levels on fruit chemistry among individuals of *Solanum carolinense*, Cipollini et al. [12] found that individuals collected in different years from locations separated by several hundred kilometers did not vary in chemical defenses of ripe fruit nor did varying levels of nitrogen and water affect variation in chemical defense. However, there are few studies examining variation of fruit toxicity within species and this warrants further investigation. Additionally, leaf extracts were only available for five species that were collected in different locations and years than fruit. Studies have shown evidence for both phenotypic plasticity [60], [61] and no phenotypic plasticity [6] in allocation to leaf defense chemistry in response to variability in available resources, and whether chemical defenses exhibit phenotypic plasticity may depend on the class of chemical compounds [60]. Whether there is intraspecific variation in the allocation of chemical defenses to fruit relative to leaves due to genetic variation or environmental factors should be further investigated to determine the generality of the results in this study.

### Conclusions

Much remains to be learned concerning the evolutionary ecology of secondary metabolites, their distribution within plant reproductive structures, and their role in mediating plant-animal and plant-microbe interactions. This study suggests that fruit toxicity against generalist natural enemies may be common in Central Panama, but that the pattern of defense varies among plant species and depends on the plant consumer. Existing studies suggest there is an adaptive value of secondary metabolites in fruit and demonstrate the multiple functions of these compounds in mediating natural enemy attack and seed dispersal [7], [13], [41], [49]. The results of this study indicate that the synergistic effects of defense compounds, along with fruit morphology, partly explain variation in predispersal seed mortality due to generalist consumers. The study of secondary metabolites and their ecological consequences is relevant not only to understanding how plants interact with their environment, but also as part of an ecological basis for drug discovery [62].

## Supporting Information

Table S1 **Summary of generalized linear mixed models for** ***Artemia franciscana*** **survivorship in fruit extract and** ***Fusarium sp.*** **hyphal growth on fruit extract relative to negative controls for mature fruit of eleven species.**
(PDF)

Table S2 **Summary of generalized linear mixed models for** ***Artemia franciscana*** **survivorship in fruit extract and** ***Fusarium sp.*** **hyphal growth on fruit extract relative to negative controls for seven species.**
(PDF)

Table S3 **Summary of generalized linear mixed model for fruit development probability in response to natural removal treatment, fruit morphology, and activity of immature fruit against** ***Artemia franciscana*** **and** ***Fusarium sp.***
(PDF)

Table S4 **Summary of generalized linear mixed model for seed germination probability in response to natural removal treatment, fruit morphology, and activity of immature fruit against** ***Artemia franciscana*** **and** ***Fusarium sp.***
(PDF)

Table S5 **Correlations between bioassays of extract activity for each plant part.**
(PDF)

Supporting Information S1 **R code for** ***a priori*** **comparisons of fungal hyphal growth and brine shrimp survivorship in response to plant extracts.**
(PDF)
