# Supplementary material for: The Distribution of Fruit and Seed Toxicity during Development for Eleven Neotropical Trees and Vines in Central Panama
Source: PLoS One. 2013 Jul 2;8(7):e66764. doi: 10.1371/journal.pone.0066764 (PMC3699617; doi:10.1371/journal.pone.0066764)
Supplement: Table S1 — Summary of generalized linear mixed models for Artemia franciscana survivorship in fruit extract and Fusarium sp. hyphal growth on fruit extract relative to negative controls for mature fruit of eleven species. (PDF) [file pone.0066764.s001.pdf]

Table S1. Summary of generalized linear mixed models for *Artemia franciscana* survivorship in fruit extract and *Fusarium sp.* hyphal growth on fruit extract relative to negative controls for mature fruit of eleven species.

**Bioassay**	**Variable**	**Estimate**	**Std. Error**	*z*
**A. *Artemia***	Intercept	-1.57	1.27	-1.24
	**Wind dispersal**	**3.74**	**1.12**	**3.33**
	**Seed**	**1.55**	**0.12**	**13.24**
	**Wind dispersal: Seed**	**-2.88**	**0.16**	**-17.62**
**B. *Fusarium***	**Intercept**	**0.79**	**0.16**	**5.03**
	Wind dispersal	0.01	0.22	0.05
	**Seed**	**0.12**	**0.04**	**3.15**
	Wind dispersal: Seed	-0.13	0.09	-1.48

Notes: The intercept is the mean of the mature pericarp from vertebrate-dispersed fruit and is the log of the odds ratio for *Artemia*. Coefficients of fixed effects are differences from the intercept. In bold are *P*-values significant at the 0.05 level.
