# Supplementary material for: The Distribution of Fruit and Seed Toxicity during Development for Eleven Neotropical Trees and Vines in Central Panama
Source: PLoS One. 2013 Jul 2;8(7):e66764. doi: 10.1371/journal.pone.0066764 (PMC3699617; doi:10.1371/journal.pone.0066764)
Supplement: Table S2 — Summary of generalized linear mixed models for Artemia franciscana survivorship in fruit extract and Fusarium sp. hyphal growth on fruit extract relative to negative controls for seven species. (PDF) [file pone.0066764.s002.pdf]

Table S2. Summary of generalized linear mixed models for *Artemia franciscana* survivorship in fruit extract and *Fusarium sp.* hyphal growth on fruit extract relative to negative controls for seven species.

Bioassay	Variable	Estimate	Std. Error	*z*
**A. *Artemia***	Intercept	-0.10	1.18	-0.09
	Wind dispersal	0.38	1.20	0.32
	Seed	0.41	0.36	0.12
	Mature	-1.83	1.36	-1.35
	**Wind dispersal: Seed**	**-0.98**	**0.34**	**-2.86**
	**Wind dispersal: Mature**	**1.94**	**0.45**	**4.34**
	Seed: Mature	0.44	0.38	1.16
	**Wind dispersal: Seed: Mature**	**-1.54**	**0.38**	**-4.01**
**B. *Fusarium***	Intercept	0.41	0.22	1.90
	Wind dispersal	0.11	0.30	0.38
	Seed	0.01	0.05	0.26
	Mature	0.17	0.12	1.43
	**Wind dispersal: Seed**	**0.39**	**0.08**	**4.62**

Notes: The intercept is the mean of the immature pericarp from vertebrate-dispersed fruit and is the log of the odds ratio for *Artemia*. Coefficients of fixed effects are differences from the intercept. In bold are *P*-values significant at the 0.05 level.
