# Supplementary material for: The Distribution of Fruit and Seed Toxicity during Development for Eleven Neotropical Trees and Vines in Central Panama
Source: PLoS One. 2013 Jul 2;8(7):e66764. doi: 10.1371/journal.pone.0066764 (PMC3699617; doi:10.1371/journal.pone.0066764)
Supplement: Table S3 — Summary of generalized linear mixed model for fruit development probability in response to natural removal treatment, fruit morphology, and activity of immature fruit against Artemia franciscana and Fusarium sp. (PDF) [file pone.0066764.s003.pdf]

Table S3. Summary of generalized linear mixed model for fruit development probability in response to natural removal treatment, fruit morphology, and activity of immature fruit against *Artemia franciscana* and *Fusarium sp.*.

Variable	Estimate	Std. Error	*z*
**Intercept**	**2.37**	**0.68**	**3.465**
**Fungicide**	**-0.69**	**0.29**	**-2.368**
Insecticide	-0.05	0.29	-0.192
Vertebrate exclosure	-0.47	0.28	-1.677
PC1	-0.18	0.12	-1.444
PC2	-0.29	0.78	-0.366
**PC3**	**0.59**	**0.24**	**2.424**
Activity of immature fruit against *Artemia*	0.23	0.34	0.666
**Activity of immature fruit against *Fusarium***	**0. 48**	**0.15**	**3.158**
**Fungicide: PC1**	**0.40**	**0.11**	**3.725**
Insecticide: PC1	0.17	0.10	1.647
**Vertebrate Exclosure: PC1**	**0.24**	**0.10**	**2.402**
Fungicide: PC2	-0.45	0.41	-1.109
Insecticide: PC2	-0.25	0.42	-0.588
Vertebrate Exclosure: PC2	-0.38	0.41	-0.928
Fungicide: PC3	0.04	0.18	0.221
Insecticide: PC3	-0.04	0.17	-0.236
Vertebrate Exclosure: PC3	0.10	0.17	0.601
**Fungicide: *Fusarium* activity**	**-0.29**	**0.11**	**-2.515**
**Insecticide: *Fusarium* activity**	**-0.24**	**0.11**	**-2.074**
**Vertebrate Exclosure: *Fusarium* activity**	**-0.33**	**0.11**	**-2.912**

Notes: The intercept is the mean (log of odds ratio) of the control group when each principal component and bioassay activity is zero. Coefficients of the treatments are differences from the control group with all PC's and bioassay activities at zero. The coefficient of each covariate, including principal components and activity against bioassays, describes the change in fruit development with one unit change in the covariate for the control group. Coefficients of interactions between treatments and covariates describe the differences in the slopes between treatments relative to the control in response to each covariate. In bold are *P*-values significant at the 0.05 level.
