# Supplementary material for: The Distribution of Fruit and Seed Toxicity during Development for Eleven Neotropical Trees and Vines in Central Panama
Source: PLoS One. 2013 Jul 2;8(7):e66764. doi: 10.1371/journal.pone.0066764 (PMC3699617; doi:10.1371/journal.pone.0066764)
Supplement: Table S4 — Summary of generalized linear mixed model for seed germination probability in response to natural removal treatment, fruit morphology, and activity of immature fruit against Artemia franciscana and Fusarium sp. (PDF) [file pone.0066764.s004.pdf]

Table S4. Summary of generalized linear mixed model for seed germination probability in response to natural removal treatment, fruit morphology, and activity of immature fruit against *Artemia franciscana* and *Fusarium sp.*

Variable	Estimate	Std. Error	*z*
Intercept	-0.51	0.57	-0.888
Fungicide	0.17	0.29	0.611
Insecticide	0.38	0.29	1.301
Vertebrate exclosure	0.14	0.28	0.515
PC1	-0.09	0.14	-0.654
**PC2**	**-0.76**	**0.25**	**-3.035**
**PC3**	**0.55**	**0.22**	**2.541**
**Activity of immature fruit against *Artemia***	**-0.50**	**0.19**	**-2.583**
**Fungicide: PC1**	**0.35**	**0.15**	**2.282**
Insecticide: PC1	0.08	0.16	0.482
Vertebrate Exclosure: PC1	0.20	0.15	1.316
Fungicide: PC2	0.07	0.22	0.298
Insecticide: PC2	0.23	0.21	1.098
Vertebrate Exclosure: PC2	0.34	0.21	1.613
Fungicide: PC3	-0.21	0.23	-0.919
Insecticide: PC3	-0.21	0.23	-0.892
Vertebrate Exclosure: PC3	-0.14	0.22	-0.633

Notes: Interpretation as for Table S3; in bold are *P*-values significant at the 0.05 level.
