# Supplementary material for: The Distribution of Fruit and Seed Toxicity during Development for Eleven Neotropical Trees and Vines in Central Panama
Source: PLoS One. 2013 Jul 2;8(7):e66764. doi: 10.1371/journal.pone.0066764 (PMC3699617; doi:10.1371/journal.pone.0066764)
Supplement: Table S5 — Correlations between bioassays of extract activity for each plant part. (PDF) [file pone.0066764.s005.pdf]

Table S5. Correlations between bioassays of extract activity for each plant part.

Plant Part	Bioassay 1	Bioassay 2	*t*	*df*	*r*	*P*
A. Immature Pericarp	*A. franciscana*	*Fusarium sp.*	-0.40	5	-0.18	0.7048
	A. franciscana	*Phoma sp.*	-0.40	5	-0.18	0.705
	Fusarium sp.	*Phoma sp.*	1.99	5	0.66	0.1034
B. Immature Seed	*A. franciscana*	*Fusarium sp.*	-0.13	5	-0.06	0.8997
	A. franciscana	*Phoma sp.*	0.80	5	0.34	0.4613
	Fusarium sp.	*Phoma sp.*	1.90	5	0.65	0.1151
C. Mature Pericarp	*A. franciscana*	*Fusarium sp.*	1.12	9	0.35	0.2928
	A. franciscana	***Phoma sp.***	**2.32**	**9**	**0.61**	**0.0458**
	Fusarium sp.	*Phoma sp.*	1.52	9	0.45	0.1631
D. Mature Seed	*A. franciscana*	*Fusarium sp.*	1.81	9	0.52	0.1032
	A. franciscana	***Phoma sp.***	**2.65**	**9**	**0.66**	**0.02639**
	Fusarium sp.	***Phoma sp.***	**3.23**	**9**	**0.73**	**0.01035**
E. Leaves	*A. franciscana*	*Fusarium sp.*	2.00	3	0.76	0.14
	A. franciscana	*Phoma sp.*	0.79	3	0.42	0.49
	Fusarium sp.	*Phoma sp.*	1.48	3	0.65	0.24
