# Supplementary material for: The Distribution of Fruit and Seed Toxicity during Development for Eleven Neotropical Trees and Vines in Central Panama
Source: PLoS One. 2013 Jul 2;8(7):e66764. doi: 10.1371/journal.pone.0066764 (PMC3699617; doi:10.1371/journal.pone.0066764)
Supplement: Supporting Information S1 — R code for a priori comparisons of fungal hyphal growth and brine shrimp survivorship in response to plant extracts. (PDF) [file pone.0066764.s006.pdf]

Supporting Information S1. R code for *a priori* comparisons of fungal hyphal growth and brine shrimp survivorship in response to plant extracts.

# Generalized linear mixed models of hyphal fungal growth with normally distributed errors

```
m1 =lmer(FusariumHyphalGrowth~Extract + (1|Dish.Nu))
m2 =lmer(PhomaHyphalGrowth~Extract + (1|Dish.Nu))
```

# Generalized linear mixed model of brine shrimp survivorship with binomially distributed errors

```
m3=lmer(cbind(Artemia.alive,Artemia.dead)~Extract+(1|Plate.Nu),family=binomial)

betahat <- fixef(m1) # Estimates of fixed effects from model
sebetahat <- sqrt(diag(vcov(m1))) # Standard errors of estimates
tval <- betahat/sebetahat # t-value
pval <- 2*pnorm(-abs(tval)) # p-value

betahat <- data.frame(betahat)
```

# Function for analysis of *a priori* contrasts

```
comparePairs=function(m,which,trmt){
	b<-fixef(m)
	s<-vcov(m)
	con<-rep(0,length(b))
	if(missing(which)){
		which<-expand.grid(1:length(b),1:length(b))
		which<-which[which[,1]!=which[,2],]
		}
		contrast<-se<-tval<-trmt1<-trmt2<-t1<-t2<-rep(0,dim(which)[1])

		for(j in 1:dim(which)[1]){
			c1<-con
			trmt1[j]=as.character(trmt[trmt[,2]==which[j,1],1])
			trmt2[j]=as.character(trmt[trmt[,2]==which[j,2],1])
			t1[j]<-which[j,1]
			t2[j]<-which[j,2]
			c1[as.numeric(which[j,])]<-c(1,-1)
			contrast[j]<-c1%*%b
			se[j]<-sqrt(c1%*%as.matrix(s)%*%c1)
			}

	data.frame(Trt1=trmt1,Trt1.nu=t1,Trt2=trmt2,Trt2.nu=t2,contrast,se,tval=tval<-
contrast/se,pval=2*pnorm(-abs(tval)))
   }
pwcomp=comparePairs(m1, w, apriori) # m1 is summary of model, w is a matrix of the contrasts,
apriori is dataframe of treatments
```
